# Hepatitis B virus Core protein nuclear interactome identifies SRSF10 as a host RNA-binding protein restricting HBV RNA production

**DOI:** 10.1371/journal.ppat.1008593

**Published:** 2020-11-12

**Authors:** Hélène Chabrolles, Héloïse Auclair, Serena Vegna, Thomas Lahlali, Caroline Pons, Maud Michelet, Yohann Couté, Lucid Belmudes, Gilliane Chadeuf, Yujin Kim, Ariel Di Bernardo, Pascal Jalaguier, François-Loïc Cosset, Floriane Fusil, Michel Rivoire, Lee D. Arnold, Uri Lopatin, Christophe Combet, Fabien Zoulim, David Grierson, Benoit Chabot, Julie Lucifora, David Durantel, Anna Salvetti

**Affiliations:** 1 INSERM, U1052, Cancer Research Center of Lyon (CRCL), Université de Lyon (UCBL1), CNRS UMR5286, Centre Léon Bérard, Lyon, France; 2 Univ. Grenoble Alpes, CEA, INSERM, IRIG, BGE, Grenoble, France; 3 INSERM U1087, Institut du Thorax, Université de Nantes, CNRS UMR6291, Nantes, France; 4 INSERM, U1111, International Center for Infectiology Research (CIRI), Université de Lyon (UCBL1), CNRS, UMR5308, Ecole Normale Supérieure de Lyon, Lyon, France; 5 INSERM U1032, Centre Léon Bérard (CLB), Lyon, France; 6 DiscoverElucidations, LLC, Rancho Santa Fe, California, United States of America; 7 Assembly Biosciences, San Francisco, California, United States of America; 8 Faculty of Pharmaceutical Sciences, University of British Columbia, Vancouver, British Columbia, Canada; 9 Department of Microbiology and Infectious Diseases, Faculty of Medicine and Health Sciences, Université de Sherbrooke, Sherbrooke, Quebec, Canada; The Pennsylvania State University College of Medicine, UNITED STATES

## Abstract

Despite the existence of a preventive vaccine, chronic infection with Hepatitis B virus (HBV) affects more than 250 million people and represents a major global cause of hepatocellular carcinoma (HCC) worldwide. Current clinical treatments, in most of cases, do not eliminate viral genome that persists as a DNA episome in the nucleus of hepatocytes and constitutes a stable template for the continuous expression of viral genes. Several studies suggest that, among viral factors, the HBV core protein (HBc), well-known for its structural role in the cytoplasm, could have critical regulatory functions in the nucleus of infected hepatocytes. To elucidate these functions, we performed a proteomic analysis of HBc-interacting host-factors in the nucleus of differentiated HepaRG, a surrogate model of human hepatocytes. The HBc interactome was found to consist primarily of RNA-binding proteins (RBPs), which are involved in various aspects of mRNA metabolism. Among them, we focused our studies on SRSF10, a RBP that was previously shown to regulate alternative splicing (AS) in a phosphorylation-dependent manner and to control stress and DNA damage responses, as well as viral replication. Functional studies combining SRSF10 knockdown and a pharmacological inhibitor of SRSF10 phosphorylation (1C8) showed that SRSF10 behaves as a restriction factor that regulates HBV RNAs levels and that its dephosphorylated form is likely responsible for the anti-viral effect. Surprisingly, neither SRSF10 knock-down nor 1C8 treatment modified the splicing of HBV RNAs but rather modulated the level of nascent HBV RNA. Altogether, our work suggests that in the nucleus of infected cells HBc interacts with multiple RBPs that regulate viral RNA metabolism. Our identification of SRSF10 as a new anti-HBV restriction factor offers new perspectives for the development of new host-targeted antiviral strategies.

## Author summary

Chronic infection with Hepatitis B virus (HBV) affects more than 250 million of people world-wide and is a major global cause of liver cancer. Current treatments lead to a significant reduction of viremia in patients. However, viral clearance is rarely obtained and the persistence of the HBV genome in the hepatocyte’s nucleus generates a stable source of viral RNAs and subsequently proteins which play important roles in immune escape mechanisms and liver disease progression. Therapies aiming at efficiently and durably eliminating viral gene expression are still required. In this study, we identified the nuclear partners of the HBV Core protein (HBc) to understand how this structural protein, responsible for capsid assembly in the cytoplasm, could also regulate viral gene expression. The HBc interactome was found to consist primarily of RNA-binding proteins (RBPs). One of these RBPs, SRSF10, was demonstrated to restrict HBV RNA levels and a drug, able to alter its phosphorylation, behaved as an antiviral compound capable of reducing viral gene expression. Altogether, this study sheds new light on novel regulatory functions of HBc and provides information relevant for the development of antiviral strategies aiming at preventing viral gene expression.

## Introduction

Despite the existence of a preventive vaccine, chronic infection with Hepatitis B virus (HBV) remains a major health problem worldwide, as it represents a major global cause of hepatocellular carcinoma (HCC) [1]. Clinically approved treatments, mainly based on nucleoside analogs (NUCs), can reduce HBV viremia under the limit of detection in patients [2]. NUCs, while potent, only affect a relatively late step in the viral life cycle, the conversion of viral pre-genomic RNA into viral DNA after encapsidation. They have no known effect elsewhere in the viral life cycle, and as a result viral clearance is rarely obtained and rebound off therapy is common, thus making life-long therapy with NUCs mandatory. The persistence of the viral genome (an episome called covalently-closed-circular dsDNA or cccDNA) in the nucleus of non-dividing hepatocytes constitutes one major obstacle toward a complete eradication of HBV infection. Indeed, cccDNA not only guarantees viral persistence in the organism but also constitutes a stable source of viral protein expression, including the HBe and HBs antigens (HBeAg and HBsAg), which play important roles in immune escape mechanisms and liver disease progression [3]. Therefore, therapies aiming at efficiently and durably blocking the production of viral antigens are still required [4,5].

HBV is a small enveloped, DNA virus that replicates in hepatocytes. After binding to its receptor, the sodium taurocholate co-transporting polypeptide (NTCP), and uncoating, the viral capsid is transported to the nucleus where the viral genome, constituted by a relaxed circular and partially dsDNA molecule of 3.2 Kb (rcDNA), is released [6]. Conversion of rcDNA into cccDNA occurs in the nucleoplasm *via* the intervention of cellular enzyme [7–9]. It results in the establishment of a viral episome that constitutes the template for the transcription of five RNAs of 3.5 (precore and pregenomic RNA), 2.4, 2.1 and 0.7 kb that, respectively, encode the HBeAg, Core protein (HBc), viral polymerase, three surface glycoproteins (S, M and L; all defining the HBsAg), and X protein (HBx). Importantly, all these RNAs are unspliced. Several other spliced RNA species are also generated. These spliced forms can be detected in the sera and livers of chronically-infected patients as well as in cells transfected with HBV genomes [10]. They are not required for virus replication but could be involved in HBV-induced pathogenesis and disease progression [11]. Formation of new viral particles initiates in the cytoplasm by packaging of the polymerase-bound pregenomic RNA (pgRNA) into the capsid. Reverse transcription of pgRNA into rcDNA occurs within capsids that are then either enveloped and secreted to form progeny viral particles or re-routed toward the nucleus to replenish the cccDNA pool [6].

HBc is the sole structural component required for the assembly of the capsid [12]. This protein of 183 amino acids (aa) is composed of a N-terminal domain (NTD, aa 1–140) that is essential for the assembly process, and a C-terminal basic domain (CTD, aa 150–183) that is dispensable for assembly. The CTD domain contains motifs responsible for trafficking of the capsid in and out of the nucleus and displays DNA/RNA binding and chaperone activities [13,14]. Studies on HBc assembly have shown that the protein forms homodimers. Capsid assembly is initiated by the slow assembly of a trimer of dimers to which HBc dimers rapidly associate to form an icosahedral capsid [12]. Packaging of Pol-pgRNA complex that occurs during capsid assembly is mediated by the CTD of HBc, which also regulates reverse-transcription of pgRNA into rcDNA [15–17].

Converging observations suggest that, besides its structural role in the cytoplasm, HBc may also exhibit important regulatory activities to control the establishment and persistence of HBV infection. First, following viral entry, HBc, derived from incoming particles, can enter the nucleus together with rcDNA, where it can form dimers/oligomers and also reassemble into “capsid-like” structures [18,19]. Nuclear entry of HBc can occur after a *de novo* infection, or as a consequence of the re-routing of capsids to the nucleus. Accordingly, nuclear HBc can easily be detected either *in vitro*, *i*.*e*. in experimentally infected human hepatocytes, or *in vivo* in the livers of chronically infected patients or model animals [20–23]. Second, earlier studies have shown that HBc binds to cccDNA, and modifies nucleosomal spacing [24,25]. Association of HBc to cccDNA was further confirmed *in vitro* and *in vivo* and correlated to an active transcriptional state [26–28]. Finally, HBc was also reported to bind to the promoter region of several cellular genes [29]. Altogether, these data strongly suggest that this structural protein may be important at some nuclear steps of the viral life cycle that remain to be clarified.

In order to gain insight into HBc nuclear functions, we performed a proteomic analysis of its cellular partners in the nucleus of human hepatocytes. Our results revealed that HBc mainly interacts with a network RNA-binding proteins (RBPs) that are involved in several post-transcriptional processes and in particular, pre-mRNA splicing. Among these RBPs, we identified SRSF10 as a host factor restricting HBV RNA synthesis/accumulation which opens new perspectives for the development of novel antiviral agents.

## Results

### Host RNA-binding proteins are major HBc interacting factors in the nucleus of differentiated hepatocytes

**Fig 1:**
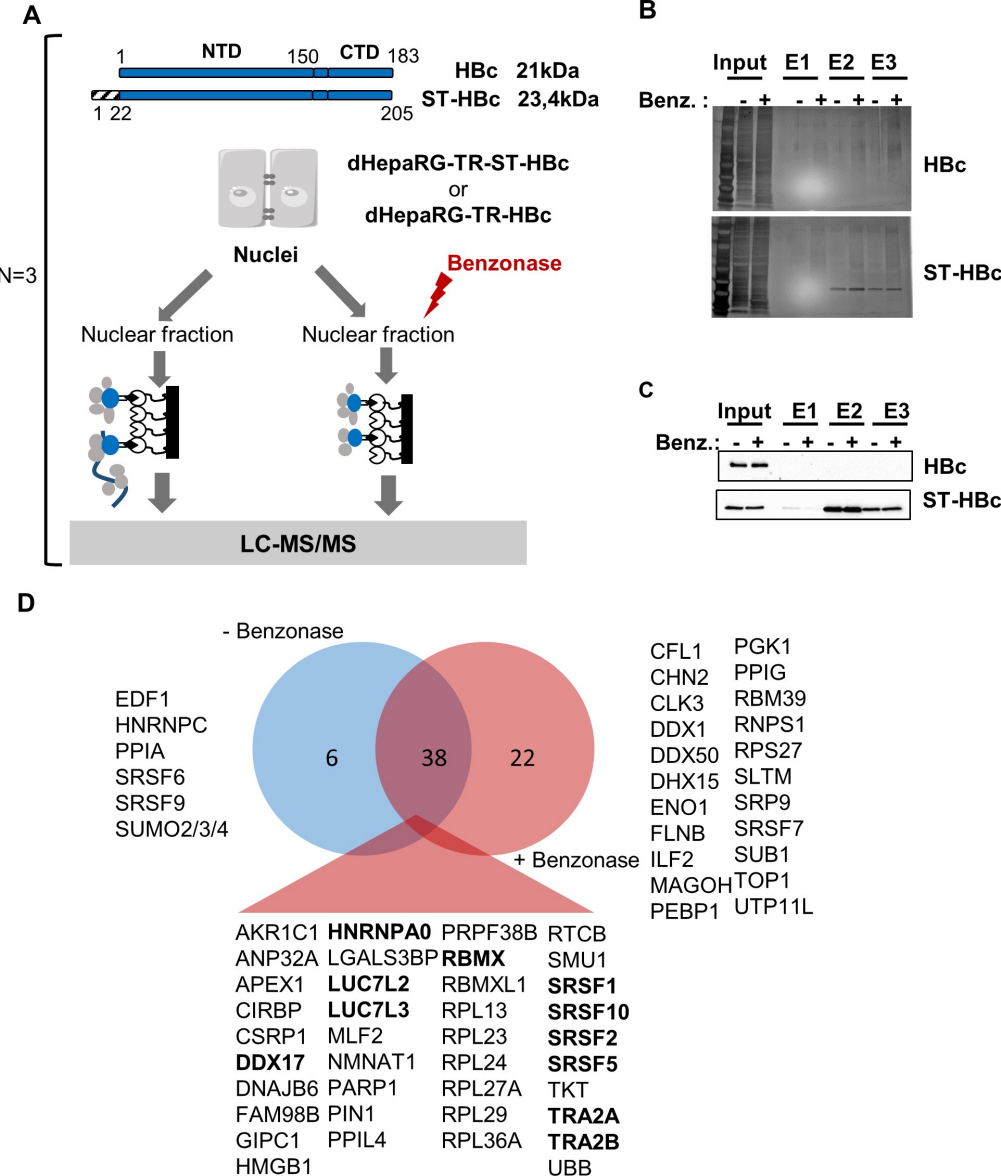
Identification of HBc-interacting proteins in the nucleus of dHepaRG cells. (A) Schematic view of HBc purification process. Nuclei were purified from differentiated HepaRG-TR cells (dHepaRG-TR) expressing either wt HBc or ST-HBc under the control of a tetracyclin-inducible promoter, lysed and then treated or not with Benzonase. Nuclear extracts were purified on a Streptactin column and protein eluted with desthiobiotin. Input and eluted fractions (E1, E2, and E3) were analyzed by gel electrophoresis followed by silver staining (B) and western blot (C) using an anti-HBc antibody. (D) Venn diagram of proteins significantly associated to HBc common to conditions with and without Benzonase. Proteins in bold correspond to the 11 “founders” RBP common to both conditions (see text, RBMXL is not highlighted because it was considered as a retrogene of RBMX).

To gain insight on HBc regulatory functions, we sought to identify its nuclear host-partners in human hepatocytes. To this end, we used differentiated HepaRG cells (dHepaRG) expressing HBc, fused at its N-terminus to a streptavidin (ST)-binding peptide (dHepaRG-TR-ST-HBc) under the control of a tetracyclin-inducible promoter (Fig 1A). The ST-HBc fusion protein localized in the nucleus of hepatocytes (S1A Fig) and assembled into capsid-like structures as wild type (wt) HBc, confirming that addition of a tag at its N-terminus did not alter these functions (S1B Fig) [30]. ST-HBc/host-factor complexes were purified from nuclear extracts on Strep-Tactin affinity columns (Fig 1B and Fig 1C). The negative control was provided by dHepaRG-TR cells expressing wt HBc, without any tag and thus unable to bind to the affinity column. In addition, to eliminate cellular partners recovered *via* DNA/RNA bridging, purification of ST-HBc-complexes was also performed on cell lysates submitted to nucleic acid digestion with Benzonase. Three independent purifications of ST-HBc-associated proteins, done with three different HepaRG differentiation batches, were performed in each condition (+/- Benzonase) and eluted proteins were analyzed by mass spectrometry (MS)-based label-free quantitative proteomics. This analysis resulted in the identification of 60 and 45 proteins found significantly associated with HBc, with and without Benzonase treatment, respectively (p-value<0.01 and fold change>4) (S1 Table). Importantly, 38 of these factors were common to both conditions, demonstrating the reliability of their identification (Fig 1D).

**Fig 2:**
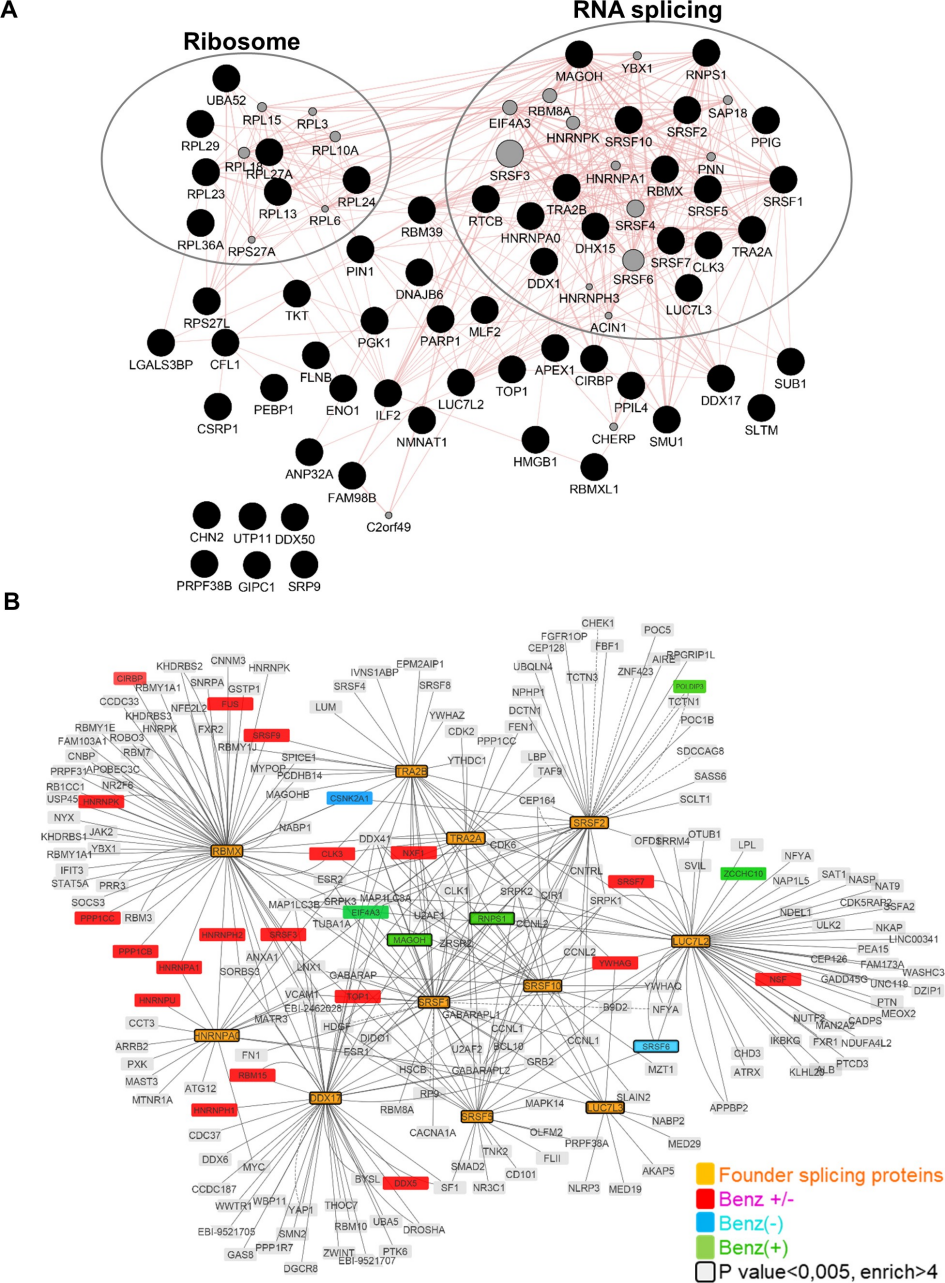
HBc nuclear interactome. (A) Proteins significantly associated to HBc in the presence of Benzonase were analyzed using the Genemania plugin in Cytoscape (3.7.1). Red lines indicate known physical interactions. Missing nodes are indicated by grey circles. (B) **Interaction network of proteins involved in mRNA splicing via spliceosome.** Significant proteins, common to the Benzonase-/+ conditions, over-representing the mRNA splicing via spliceosome biological process (“founder proteins” highlighted as orange nodes) were used to initiate the network by querying IntAct database. Red, blue and green nodes denote protein of the computed network that are found in the proteomic hits of both Benzonase-/+ (Benz- or Benz+) conditions. Nodes with a bold border indicate significant proteins (p-value<0.005 and fold change>4) from the proteomics data.

Gene ontology (GO) annotation of HBc-interacting factors, revealed that approximately 50% of the factors, identified with or without Benzonase treatment and significantly associated with HBc, were nucleic acid binding proteins and belonged to the RBP family. In the presence of Benzonase, the most abundant protein category (Q-value: 1.8 x10^-29^) identified, corresponded to factors involved in RNA post-transcriptional processes, in particular splicing (Fig 2A). The second most-relevant category (Q-value: 4.4x10^-14^) corresponded to ribosomal proteins. The similarity of the interactome obtained with and without Benzonase suggests that most of these interactions occurred in the absence of nucleic acids or, alternatively, that they formed high order complexes in which the DNA/RNA was protected from nuclease digestion.

As the major GO category corresponded to RBPs involved in splicing, we next focused on proteins corresponding to this functional group and common to conditions with and without Benzonase (*i*.*e*. 11 proteins highlighted in bold in Fig 1D). The interactome of these 11 RBPs, hereafter designed as “founder” RBPs, showed that they were highly inter-connected and that several of their first-level interacting partners were also found among HBc-co-purified factors (Fig 2B).

**Fig 3:**
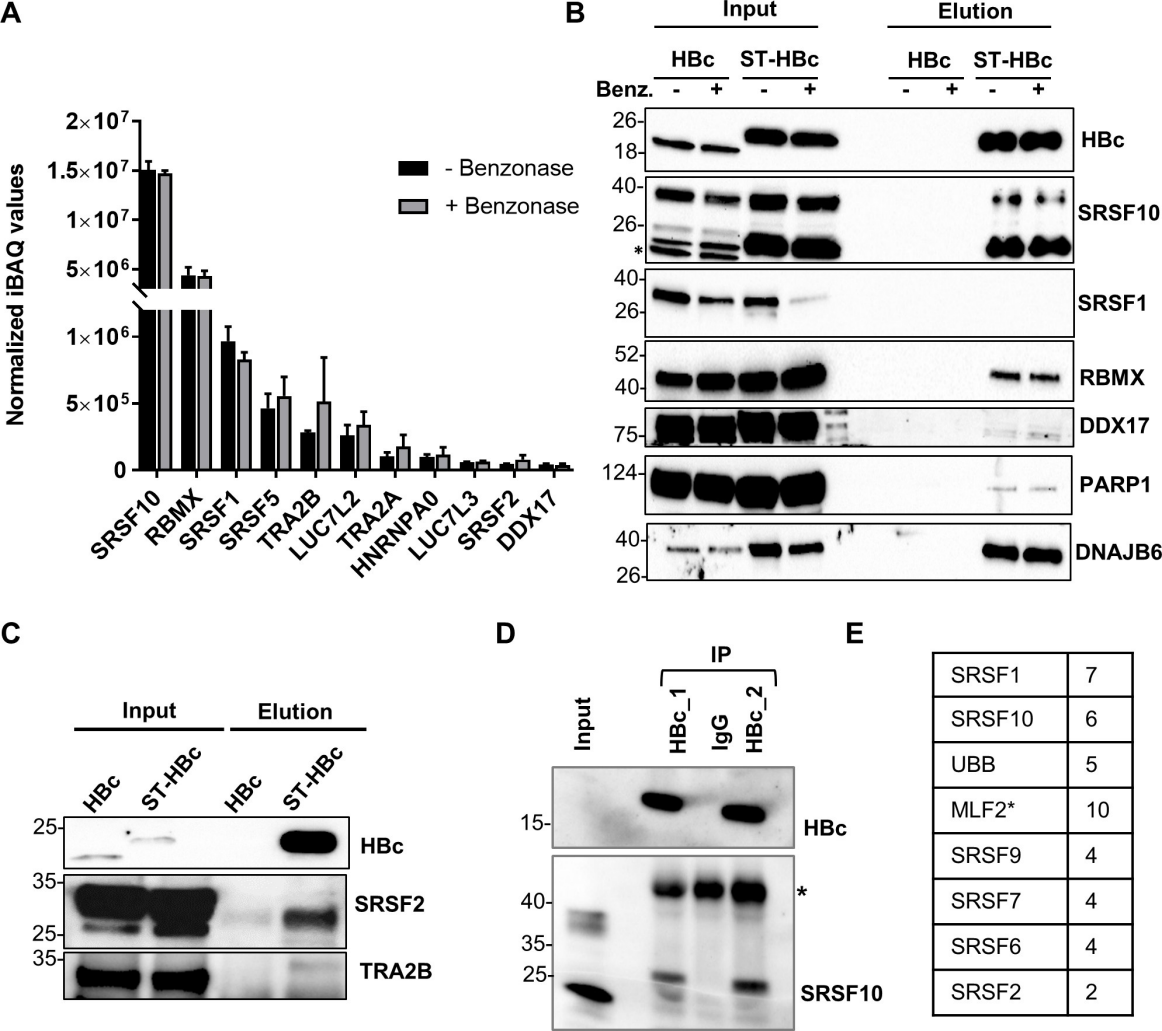
Validation analyses. (A) Relative abundances of the 11”founder” RBPs identified in HBc nuclear complexes submitted or not to Benzonase treatment. The relative abundances of HBc binding partners have been evaluated using the iBAQ metrics [104]. For each replicate, each iBAQ value was normalized by the summed values of the 11 proteins. Error bars represent +/- SD. (B) and (C) Western blot validations in Benzonase treated and streptactin-purified and extracts. Two major isoforms of SRSF10 are visible: the upper at 37KDa and the lower at 20–22 KDa. The band indicated with an asterisk likely corresponds to a band generated by proteolytic cleavage. (D) HBc was immune-precipitated from nuclear extracts purified from liver sections from HBV-infected HuHep mice, using two different anti-HBc antibodies. Eluted proteins were analyzed by western blot using anti HBc and anti-SRSF10 antibodies. The asterisk indicates the positions of IgG heavy chain. (E) Proteins included in the gel band between 35 and 25 KDa were analyzed by MS. The table indicates the list of proteins recovered with the anti-HBc antibody that were also previously found after HBc purification on StrepTactin columns (see Fig 1D). * In the case of MLF, 1 peptide was found in the anti-IgG control IP. The other proteins were found exclusively in the anti-HBc IP.

The analysis of the relative abundance of these founder RBPs indicated that SRSF10 was the most abundant RBPs co-purified in HBc-complexes, followed by RBMX, SRSF1, SRSF5 and TRA2B (Fig 3A). Western blot analyses confirmed the presence of SRSF10, RBMX, DDX17, SRSF2 and TRA2B in ST-HBc purified complexes, as well as that of two other non-RBP factors, PARP1 and DNAJB6 (Fig 3B and 3C). In contrast, the presence of SRSF1 could not be confirmed by Western blot (Fig 3B). The reason for this lack of detection is presently unclear but it could be due to a poor sensitivity of the antibodies used.

### HBc interacts with multiple SRSF10 isoforms

SRSF10, the most abundant cellular protein in our HBc nuclear interactome study, is a member of the SR protein family of splicing factors [31,32]. As all the other members of the SR family, SRSF10 is composed of a N-terminal RNA recognition motif (RRM) and a C-terminal arginine an serine-rich domain (RS) that is responsible for binding to other RBPs and that, in the case of SRSF10 is split in two modules, RS1 and RS2 [33]. Two isoforms of SRSF10 of 37 and 20 KDa have been originally described, the smaller presenting a deletion of the C-terminal RS2 domain, but only the full-length has been extensively studied (S2A Fig). The confirmation of the interaction between HBc and SRSF10 in an infectious cell culture model was extensively studied by co-immunoprecipitation. Using *in vitro* HBV-infected dHepaRG or freshly isolated primary human hepatocytes (PHH), results were inconclusive, maybe due to the rather low levels of infection in these models. In order to ascertain the interaction between HBc and SRSF10, co-immunoprecipitation (co-IP) analyses were performed using hepatocytes from mice engrafted with human hepatocytes and infected with HBV (HuHep mice), a model in which the replication level of HBV is very high. Using these samples, we found that IP of HBc, performed using two different antibodies led to detection of a band reacting with the anti-SRSF10 antibody but migrating at a size between 25 and 35 KDa that did not correspond to the size of the two major SRSF10 isoforms of 37 and 20 KDa detected in the input fraction (Fig 3D). Because this size was unexpected, proteins present in the gel band included between 35 and 25 KDa were analyzed by MS. This analysis confirmed the presence of SRSF10 that was identified by 6 different peptides, covering the first 100 aa, found exclusively in the anti-HBc sample (Fig 3E). In addition to SRSF10, seven other proteins, among which five SR proteins, previously identified in the ST-HBc eluted fraction (Fig 1D) were also detected. Importantly, coIP performed using dHepaRG-HBc or ST-HBc nuclear extracts similarly detected this new SRSF10 band in addition to the other conventional SRSF10 isoforms (S2B Fig). At least 9 different SRSF10 isoforms, all containing the RRM and RS1 domains, are predicted to be produced from alternatively spliced transcripts (https://www.ncbi.nlm.nih.gov/gene/10772). This new under-represented isoform of approximately 30–32 KDa may correspond to a variant of 217 aa (S2C Fig) that contains the RRM and RS1 domains, as well as a shorter RS2 domain (NP_001300937.2).

Altogether, these results indicated that the interaction between HBc and SRSF10 as well as with other, previously identified SR proteins was maintained in HBV-infected PHH. It also indicated that HBc can potentially associate with different SRSF10 isoforms further suggesting the potential importance of this interaction for the viral life cycle.

### SRSF10 modulates HBV RNA levels

**Fig 4:**
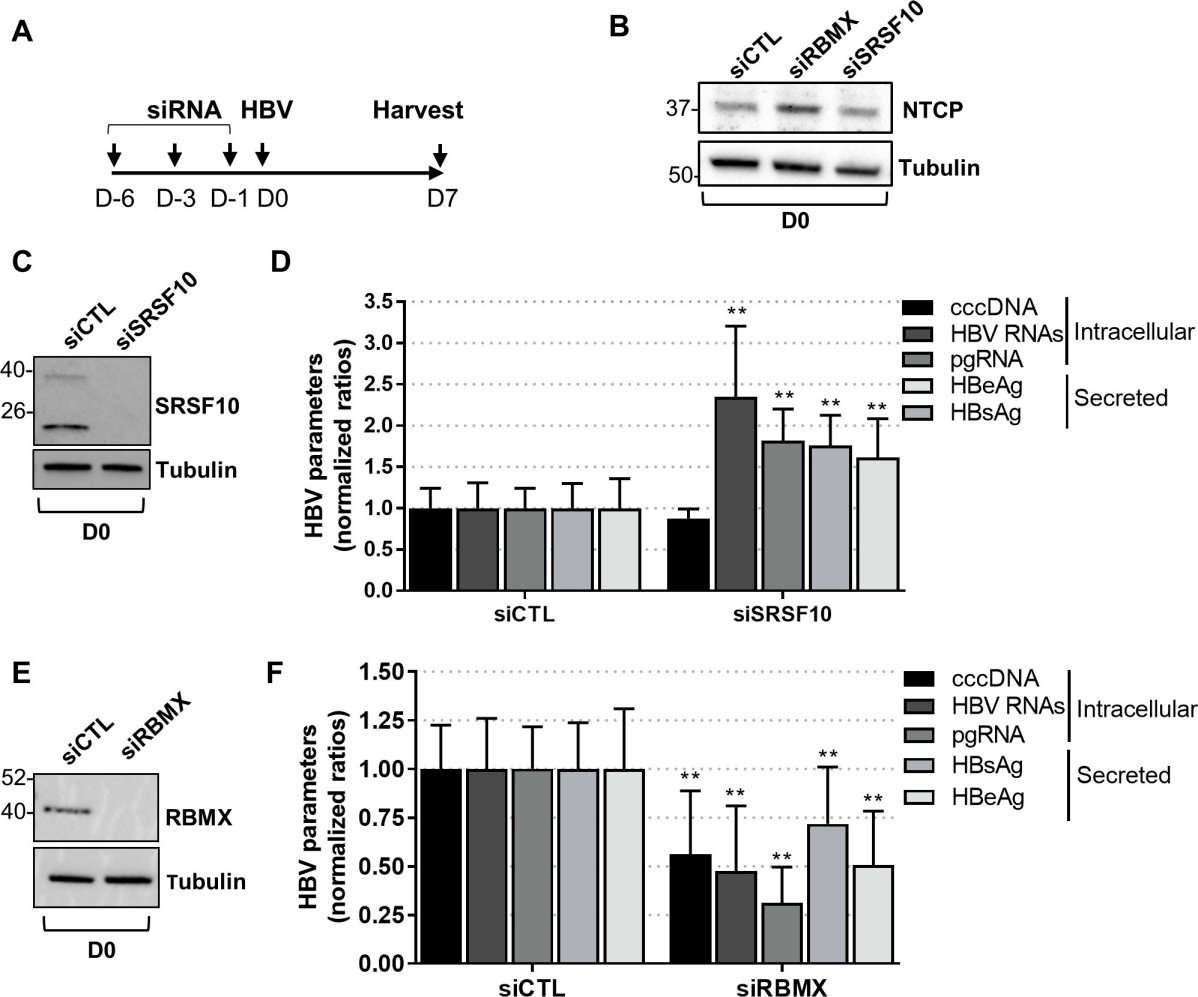
Effect of SRSF10 or RBMX KD on HBV replication in PHH. (A) Outline of the experimental protocol: cells were transfected with siRNA targeting SRSF10 or RBMX or control siRNA (siCTL) and then infected with HBV (MOI of 100 vge/cell). Cells and supernatants were harvested 7 days (D) post-infection (pi) and analyzed to measure intracellular and secreted HBV parameters. (B), (C) and (E) Western bot analysis of NTCP, SRSF10 and RBMX protein levels at D0 of the protocol, respectively. (D) and (F) HBV intracellular and extracellular parameters were measured at D7 pi. Results are expressed as the mean normalized ratio +/- SD, between siSRSF10 or siRBMX and siCTL transfected cells, of 3 independent experiments, each performed in triplicate, with PHH from different donors.

We next investigated the effect of a SRSF10 knock-down (KD) on HBV infection. Importantly, the siRNAs used to KD SRSF10 localized to the RRM and RS1 coding sequence and thus potentially targeted all the SRSF10 isoforms, including those not visible by Western blot, since they all share these domains (S2A Fig). Optimization of the siRNA transfection protocol led to a significant level of protein KD in PHH without affecting NTCP levels, strongly suggesting that HBV internalization was not affected (Fig 4A–4C). In PHH, KD of SRSF10 resulted in a significant increased accumulation of total HBV RNAs and pgRNA without affecting cccDNA level (Fig 4D). Similar results were observed in dHepaRG cells with the exception that, in this cell model, a modest but significant increase in cccDNA level, was observed (S3A–S3D Fig). The effect of SRSF10 KD on HBV RNAs was also confirmed by Northern blot confirming the increase of the three detectable HBV RNAs molecules (S4 Fig). In sharp contrast, KD of RBMX resulted in opposite effects on HBV replication, with a decrease of all viral parameters, including cccDNA (Fig 4E and 4F, S3 Fig). These results indicated that RBPs found associated with HBc play distinct roles in the HBV life cycle. To determine whether SRSF10 KD had similar effect on an already established HBV infection, siRNA-mediated KD was also performed 7 days after the onset of infection, when replication has reached a plateau [34,35]. In dHepaRG cells, a reproducible increase of HBV RNA could be observed following SRSF10 KD even if at a lower level as compared to cells in which the KD was performed before infection (S5 Fig). As previously observed in cells transfected before infection, SRSF10 KD also increased cccDNA level suggesting that, in this cell type, SRSF10 may modulate cccDNA recycling and /or stability in addition to its effect on HBV RNAs.

Finally, to investigate if the SRSF10 effect on HBV RNAs was dependent on HBc, we used AAV vectors to deliver into hepatocytes either a wt or mutated HBV genome unable to produce HBc and compared their replication level in the presence in the absence of SRSF10 (S6A Fig). In particular two mutated AAV-HBV genomes were used: one with a point mutation of the HBc ATG (AAVHBVnoHBc), and the other with a 406 bp deletion of the HBc coding sequence included between the HBc ATG and the beginning of the polymerase ORF (AAVHBVΔHBc). Transduction of dHepaRG cells with these three vectors led to the establishment of HBV infection as detected by the quantification of HBV RNAs and secreted antigens (S6B–S6D Fig). As expected the AAVHBVnoHBc vector produced both HBs and HBeAg whereas only HBsAg was detected using AAVHBVΔHBc-transduced cells. KD of SRSF10 increased all viral parameters in AAVHBVwt-transduced cells. Interestingly, the increase in HBV RNAs and secreted antigens measured following KD of SRSF10 was significantly reduced in the absence of HBc suggesting that the SRSF10 anti-viral effect may be partially dependent on HBc.

Altogether these results indicate that SRSF10 behaves as a restriction factor that mainly modulates HBV RNA levels.

### A small molecule inhibitor of SRSF10 phosphorylation strongly impairs HBV replication and antigen secretion

**Fig 5:**
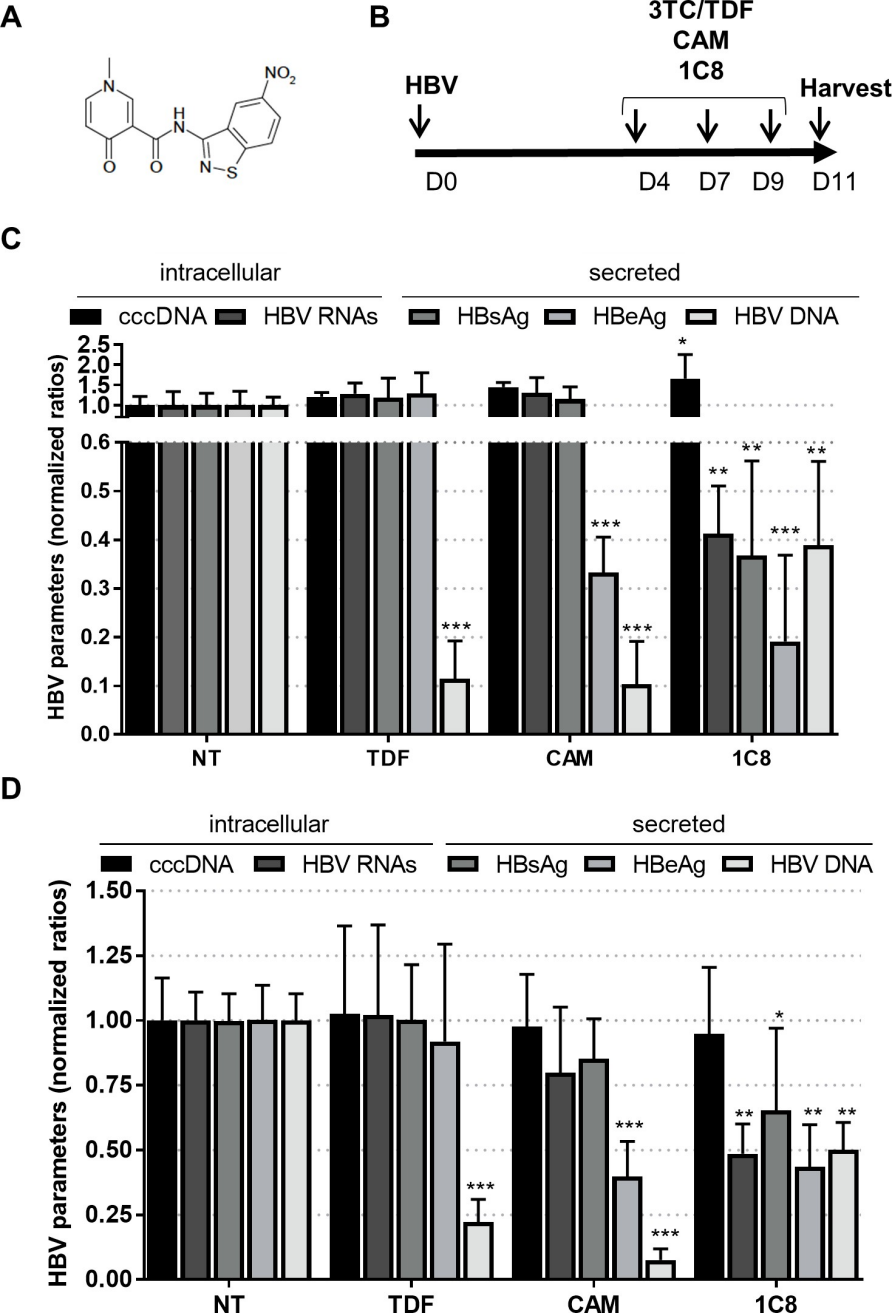
Effect of 1C8 on an established HBV infection. (A) Molecular structure of 1C8. (B) Outline of the experimental protocol: HBV-infected dHepaRG cells (C) or PHH (D) were treated three times with Tenofovir (TDF at 10μM), a Core allosteric modulator (CAM at 10μM) or 1C8 (10 μM) starting at D4pi. Intracellular and secreted HBV parameters were quantified 2 days after the last treatment. Results are expressed as the mean normalized ratio +/- SD between non-treated and treated cells of 3 independent experiments, each performed in triplicate.

SRSF10 activity was previously shown to be tightly controlled by phosphorylation, which regulates its interaction with other RBPs and splicing activities [36–39]. De-phosphorylation of SRSF10 occurs in response to heat shocks, DNA damage or during mitosis. More recently, compound 1C8 (Fig 5A), was shown to prevent SRSF10 phosphorylation, in particular at serine 133, in the absence of any other detectable effect on other SR proteins, and to inhibit HIV-1 replication with a combined effect on HIV-1 transcription and splice site selection likely producing an imbalance in viral protein required for replication [40,41].

Using a two-dimensional gel electrophoresis, we confirmed that 1C8 could induce the dephosphorylation of SRSF10 in differentiated human hepatocytes (S7 Fig). To explore the effect of 1C8 on HBV replication we first assessed its effects when added on HBV-infected dHepaRG cells (Fig 5B). In this setting, treatment with 1C8 resulted in a decrease of viral RNAs and all downstream secreted parameters (Fig 5C). Interestingly, this phenotype was different from that observed with other antiviral compounds such as a NUC (Tenofovir) that uniquely inhibited HBV DNA synthesis, or a Core allosteric modulator (CAM) that additionally inhibited HBe secretion [42]. This effect, although weaker, was maintained in HBV-infected PHH (Fig 5D), a more relevant/physiologic model to assess the activity of compounds targeting host functions. Dose response analyses in dHepaRG indicated an effective concentration 50% (EC50) of approximately 10 and 5 μM for HBV RNAs/secreted DNA and HBsAg/HBeAg, respectively, in the absence of detectable cell cytotoxicity (S8 Fig). In subsequent analyses we sought to determine if 1C8 was equally active on other HBV genotypes than D that was used in all our previous experiments. In dHepaRG cells we found that 1C8 could inhibit the replication of HBV genotype C, with a significant decrease of viral RNAs and all secreted parameters (S9A Fig). A preliminary analysis with five other HBV genotypes also indicated that 1C8 could significantly reduce HBS and HBeAg secretion in particular for genotypes G and H, suggesting that its effect may be pan-genotypic (S9B Fig).

These results indicate that 1C8 can inhibit HBV replication by reducing HBV RNA levels. The inhibitory effect of 1C8 on HBV RNAs, opposite to that observed following SRSF10 KD, suggests that the de-phosphorylated form of SRSF10, that is depleted following siRNA transfection and, in contrast, induced after 1C8 treatment, is responsible for the observed antiviral activities of this cellular RBP.

### 1C8 antiviral effect is partially dependent on SRSF10, promoting a reduction in HBV RNAs but not their splicing

**Fig 6:**
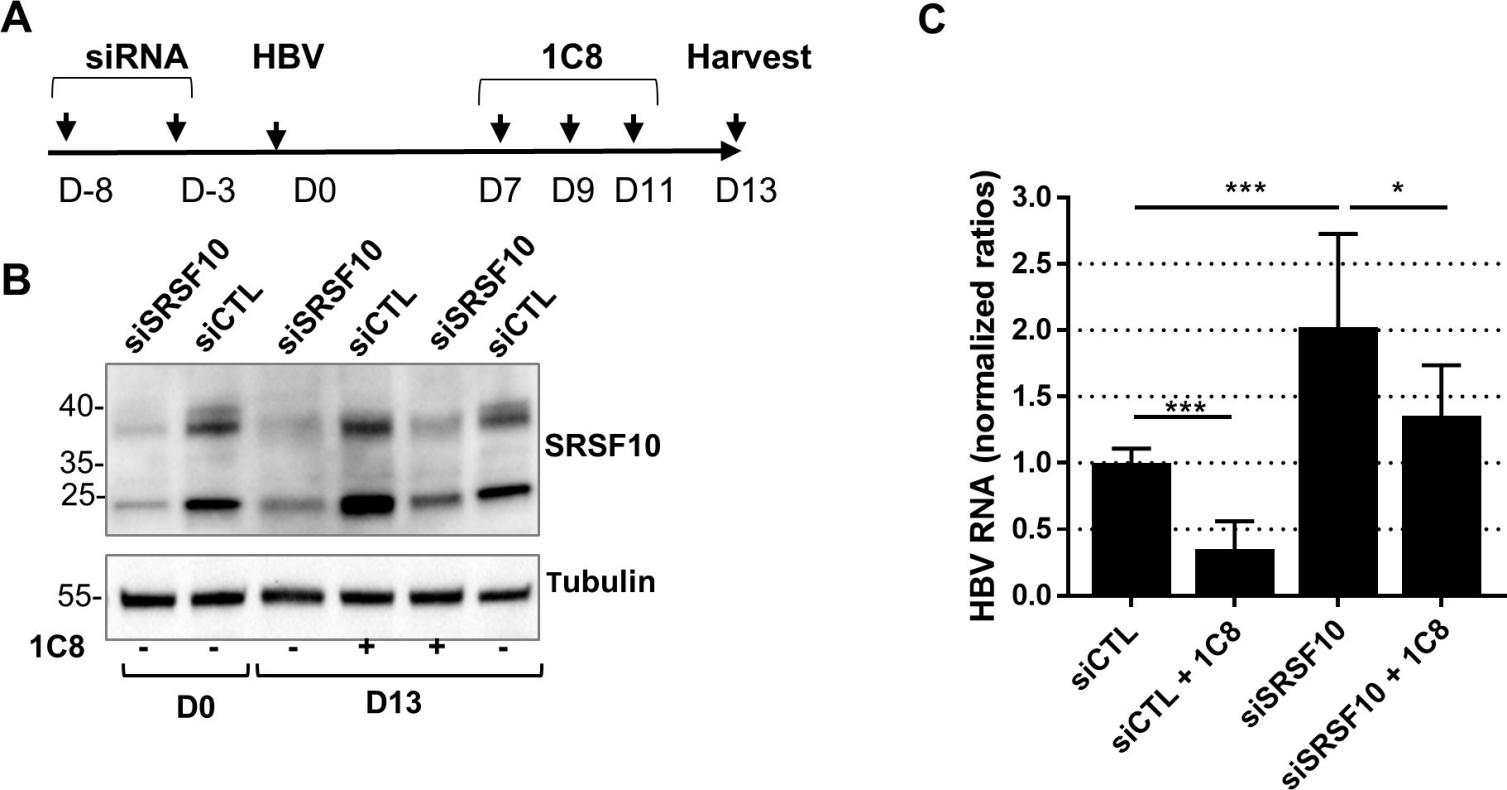
Combined effect of sRSF10 KD and 1C8 treatment on HBV-infected dHepaRG cells. (A) Outline of the experimental procedure: dHepaRG cells were transfected once or twice with siRNA targeting SRSF10, then infected with HBV (MOI of 250 vge/cell), and 7 days later treated three times with 1C8 (10μM). (B) Western blot validation showing SRSF10 depletion. (C) Quantification of intracellular HBV RNAs. Results are expressed as the mean normalized ratio +/- SD between treated and/or siSRSF10 transfected cells and siCTL-transfected cells of 3 independent experiments, each performed in triplicate.

To verify if the effect of 1C8 on HBV RNA accumulation was indeed related to SRSF10, experiments combining SRSF10 KD and 1C8 treatment were conducted (Fig 6A). Based on the model proposed, the inhibitory effect of 1C8 on viral RNA production, should be prevented by depleting SRSF10. As previously observed, each treatment alone, 1C8 or siSRSF10, resulted in opposite effects on HBV RNA levels. Remarkably, in cells receiving both treatments, depletion of SRSF10 could partially rescue the inhibitory effect of 1C8 to a level similar to that observed in control cells without, however, reaching that measured in siSRSF10-transfected cells (Fig 6B and 6C). This result indicates that the antiviral effect of 1C8 is dependent on SRSF10. The lack of a complete rescue, in cells treated with 1C8 and depleted of SRSF10, could be explained by the persistence of a low level of dephosphorylated SRSF10. Alternatively, it is possible that 1C8 additionally targets other cellular and/viral factors that are involved in the anti-viral effect.

**Fig 7:**
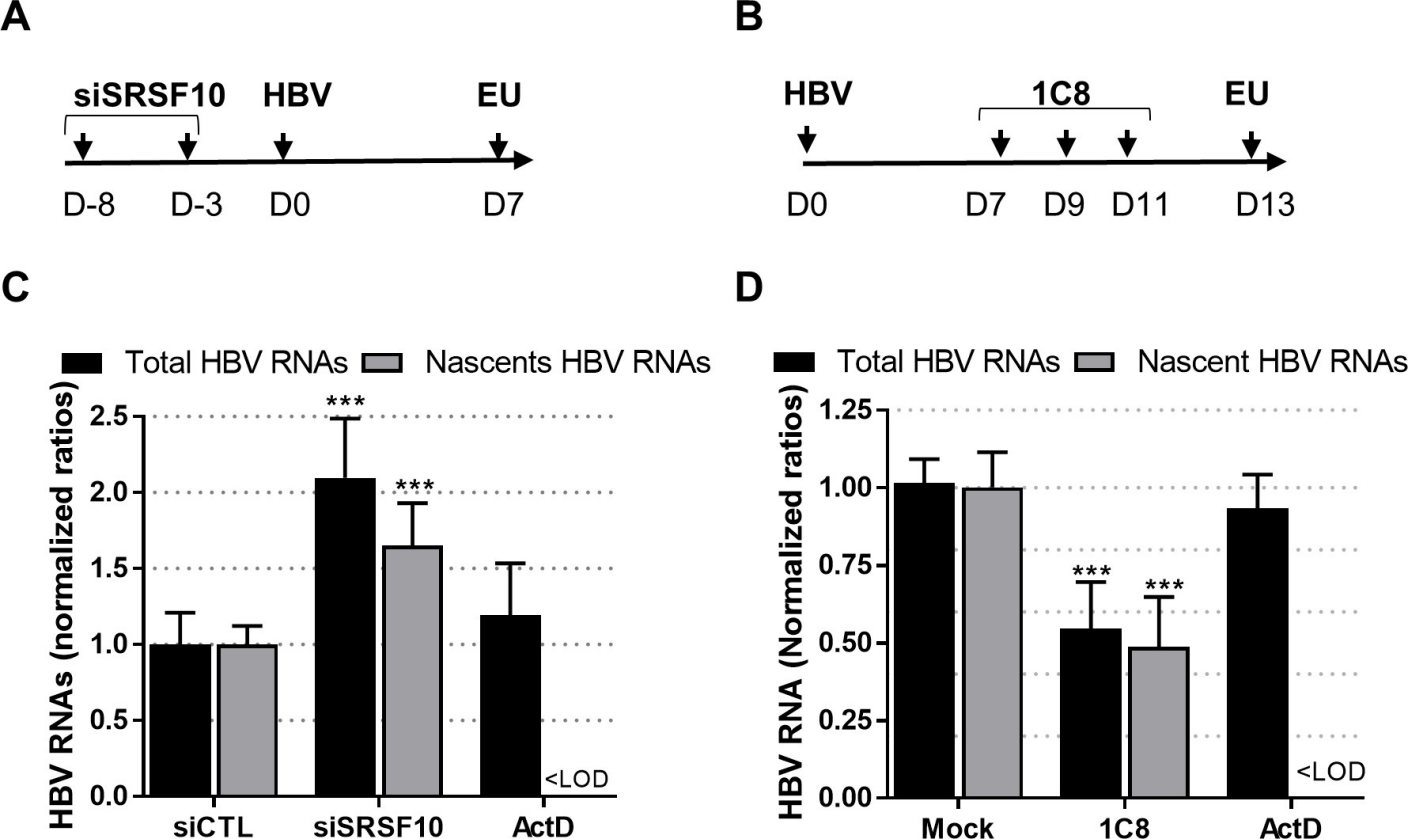
Analysis of nascent HBV RNAs following SRSF10 KD or 1C8 treatment. (A) dHepaRG cells were transfected with siRNA against SRSF10 and then infected with HBV (MOI of 250 vge/cell). Edu labelling was performed at D7pi for 2 hours. (B) dHepaRG cells were infected with HBV and then treated three times with 1C8 (40μM) at D7, D9 and D11pi. EU incorporation was performed at D13pi for 2 hours. (C) and (D) Run-on analyses. Intracellular RNA was extracted from transfected/treated cell cells and either directly quantified using HBV primers (Total HBV RNAs) or purified using the Click-iT Nascent RNA Capture kit to quantify newly synthetized RNAs (nascent HBV RNAs). Control was provided by treating cells with Actinomycin D (ActD at 10mg/ml) added to cells 20 min before labeling (see Methods). <LOD: under the limit of detection.

All HBV RNAs required and sufficient for a productive replication (*i*.*e*. production of virion and viral proteins) are unspliced. Nonetheless, several spliced HBV mRNA have been documented in experimental models and more importantly in patient samples, indicating that, if an active mechanism of escape from splicing exists, it must be partial and/or ineffective at a certain stage during chronic infection [10]. Among the numerous HBV spliced RNAs, two major spliced forms result in the production of new viral proteins, some being potentially involved in viral pathogenesis, and particles containing shorter viral genomes [10,43,44]. In our previous assays, the primers used to quantify HBV RNAs (total and pgRNA) localized to an unspliced region of the HBV genome. Therefore, it was possible that the variations in HBV RNA levels observed after SRSF10 KD or 1C8 treatment could be due to a specific modulation in some spliced variants or to a differential effect on spliced versus unspliced forms. To explore this possibility RNA extracted from siRNA transfected hepatocytes were analyzed by RT-qPCR using primers able to specifically detect each spliced and unspliced RNA (S2 Table). Unexpectedly, the relative quantification of each RNA variant in SRSF10-depleted versus control cells resulted in a global increase of all HBV RNA variants, in particular in PHH, including all detected spliced forms without inducing a preferential modulation of a spliced versus unspliced variants (S10A and S10B Fig). Similarly, treatment of HBV-infected dHepaRG cells with 1C8 post-infection resulted in a strong reduction of all viral RNA whether spliced or unspliced (S10C Fig). These results indicated that the respective proviral or antiviral effect of SRSF10 KD or 1C8 was not associated to a variation in the level of spliced versus unspliced HBV RNAs. They also suggested that both treatments acted on HBV RNAs synthesis and/or stability. To verify this point, total and nascent HBV RNAs were quantified following SRSF10 KD or 1C8 treatment. The quantification of nascent HBV RNAs was performed by labeling newly transcribed RNAs with ethynyl uridine (EU) for 2 hours before capture (Fig 7A and 7B). As expected, depletion of SRSF10 in dHepaRG cells prior to HBV infection increased total HBV RNAs. Actinomycin D (ActD), a global transcription inhibitor, strongly reduced the level of nascent RNA. In contrast, in cells transfected with siSRSF10, newly transcribed HBV RNAs were increased at a level similar to that observed for total RNAs (Fig 7C). The same analysis performed on 1C8-treated cells indicated that the compound equally reduced total and nascent HBV RNAs (Fig 7D). Altogether, these analyses indicate that SRSF10 and 1C8 did not modify the splicing level of HBV RNAs but, rather, that both treatments exert their effect by modifying the transcription and/or the stability of nascent viral RNAs.

## Discussion

Our strategy to expand the knowledge on HBc nuclear functions was based on the identification of its host protein partners in the nucleus of dHepaRG cells, when expressed alone in the absence of other viral constituents. HepaRG cells represented one of the best alternative models to freshly isolated human hepatocytes, and could be easily engineered as opposed to PHH [45]. This analysis indicated that in the nucleus HBc interacts with RBPs that are mainly involved in all steps of mRNA metabolism (Fig 2). Importantly, some of these factors were equally identified as HBc-interacting partners in hepatocytes from HBV-infected HuHep mice (Fig 3E). Therefore, even if most of these interactions need to be further validated in other relevant models, the results of both analyses suggest that these cellular RBPs play a role during viral replication.

Most of these RBPs localize in well-defined nuclear bodies, in particular speckles and paraspeckles [46,47]. Recent studies have highlighted that many active genes are located in proximity to nuclear speckles and that this association results in an increase of nascent transcript levels [48,49]. The association of HBc with cellular RBPs strongly suggests that it may intervene in the metabolism of viral RNAs by interacting with a highly interconnected network of proteins that can act at several transcriptional and post-transcriptional steps [50]. Accordingly, HBc has several features similar to those found in many cellular RBPs with, notably, a positively-charged CTD composed by a long stretch of arginines separated by seven serine residues resembling the arginine/serine-rich domain (RS domain) of several RBPs in particular of SR proteins [17,51,52]. When expressed in bacteria, HBc, thanks to its CTD, displays a strong RNA-binding activity [53]. Interestingly, several studies have shown that HBc also has a strong affinity for DNA, and can associate with cccDNA both *in vitro* and *in vivo* [24–27]. Although no evidence yet support the binding of HBc to viral and/or cellular RNA in the nucleus of HBV-infected hepatocytes, HBc, like some RBPs, may possess the dual ability to bind to DNA and RNA [54]. This activity might be tightly modulated by the phosphorylation level of its CTD, as previously demonstrated during pgRNA packaging [17]. Alternatively, the association of HBc with cccDNA and/or RNA may be indirectly mediated *via* RNA molecules and/or its interaction with cellular RBPs. Interestingly, some RBPs found associated to HBc, such as RBMX (Fig 1) were also reported to bind to DNA, in particular during DNA repair [55,56].

Among the RBPs interacting with HBc, we focused on SRSF10, a member of the SR proteins family that was the most highly abundant in HBc-containing complexes. Initial studies identified SRSF10 as a splicing repressor when de-phosphorylated in response to heat shock [39,57]. Further analyses showed that SRSF10 is a regulator of AS, depending on its phosphorylation level that also determines its interaction with diverse RBPs, in particular TRA2A, TRA2B, hnRNPK, F, and H [36,39,58]. Importantly, SRSF10 influences the AS of several cellular transcripts involved in pathways of stress response, DNA damage response, apoptosis, and carcinogenesis [58–60]. In accordance to its role in the stress response, SRSF10 was described as a component of paraspeckles, nuclear stress bodies and cytoplasmic stress granules [61–63]. SRSF10 also plays a role in the control of viral RNA, in particular of HIV-1 [41,64]. As all the other members of the SR family, SRSF10 is composed of a N-terminal RNA recognition motif (RRM) and a C-terminal RS domain that is responsible for binding to other RBPs [33]. Two isoforms of SRSF10 of 37 and 20 KDa have been originally described corresponding to those detected by available antibodies, the smaller presenting a deletion of the C-terminal domain (S2A Fig). In co-IP analyses performed on liver samples from HBV-infected HUHep mice, we found that HBc mainly interacted with a new SRSF10 isoform with an apparent molecular weight of 32KDa that was equally visible when co-IP was performed on nuclear extracts from HBc-expressing dHepaRG cells (S2B Fig). The identity of this new isoform is presently unclear but it may correspond to one of the 9 protein variants that can be generated by AS of the SRSF10 pre-mRNA. Why this isoform was detected only after IP and not following purification on StrepTactin columns (Fig 3B) is presently unclear. One hypothesis is that HBc may differentially interact with various SRSF10 isoforms according to its assembly state (dimer, oligomer or capsid-like structures), and that the ratio of these HBc forms may vary depending on the purification procedure.

To investigate the role of SRSF10 during HBV replication we analyzed the consequences of its KD in differentiated hepatocytes. Depletion of SRSF10 induced a reproducible increase in viral RNAs, proteins and secreted DNA that was observed in both PHH and dHepaRG (Fig 4 and S3 Fig). These results suggest that SRSF10 may normally repress the production of viral RNAs. While our results show that HBc and SRSF10 interact with each other, the role of this interaction in the regulation of the overall viral RNA amount remains unclear. In particular, future studies should be performed to decipher the role of HBc in the nuclear biogenesis of viral RNAs and to determine if and how it may counteract SRSF10 restrictive activity. The importance of SRSF10 in the HBV life cycle was further suggested by the finding that compound 1C8, previously characterized as an inhibitor of SRSF10 phosphorylation [41], inhibited HBV replication by inducing a marked reduction of viral RNA levels in both HBV-infected dHepaRG cells and PHH (Fig 5). The opposite effects observed upon SRSF10 depletion and 1C8 treatment strongly suggest that the de-phosphorylated form of SRSF10 is responsible for the restriction effect. Accordingly, we confirmed that 1C8 equally induced SRSF10 de-phosphorylation in dHepaRG cells (S7 Fig). Following to this hypothesis, while treatment with 1C8 results in the accumulation of de-phosphorylated SRSF10, leading to a strong inhibitory phenotype, depletion of SRSF10 using siRNA affects all SRSF10 forms whether phosphorylated or not, thereby attenuating its restrictive activity. The involvement of SRSF10 in 1C8 antiviral effect is further suggested by the finding that it could be partially reverted upon SRSF10 depletion. As previously shown during AS, de-phosphorylation of SRSF10 may change its interaction with other cellular proteins important for HBV life cycle as well with HBc finally affecting its capacity to bind viral RNA [39,41]. It is also possible that 1C8 prevents the phosphorylation of other targets in addition to SRSF10. In particular, 1C8 may interfere with the phosphorylation of HBc in the nucleus, with consequences on its capacity to bind not only to other factors, in particular RBPs, but also DNA and/or RNA. Phosphorylation of HBc at serine residues within its CTD has been described as an important event to regulate pgRNA packaging and reverse transcription during nucleocapsid formation in the cytoplasm [17,65–69]. An attractive hypothesis is that phosphorylation of the nuclear pool of HBc may similarly control its capacity to bind to DNA and/or RNA by modulating its positive charges, as previously demonstrated by studies performed in bacteria [53]. The identification of kinase(s) inhibited by 1C8 will be critical to further decipher its mode of action and develop new, more efficient and selective compounds that could be used in combination with other already in use anti-viral drugs (such as NUC or CAM) to induce a long-lasting inhibition of viral replication. Of course, this perspective should first tackle the potential toxicity issue raised by using a host-targeting agent.

Finally, one surprising finding of our study is that neither SRSF10 nor 1C8 altered the splicing of HBV RNAs, as compared to what observed for other viruses (S10 Fig) [41,70]. While this result does not exclude that part of their effects on HBV may be indirectly mediated by an alteration in splicing of other cellular RNAs, our data rather suggest that both SRSF10 and 1C8 may act by controlling the level of nascent HBV RNAs (Fig 7). Beside their canonical role during splicing, several SR proteins were found to act at several other steps of cellular and viral RNA metabolism including transcription, stability and nuclear egress [32,71,72]. Notably, besides pre-mRNA splicing, some SR proteins have been reported to directly or indirectly associate to the phosphorylated CTD of RNA polymerase II and to stimulate transcriptional elongation [73,74]. Interestingly, several RBPs previously identified as important for HBV replication were shown to participate in the control of HBV transcription and/or in RNA stability. This is the case in particular for the splicing factors HNRNPC, HNRNPK, PUF60, RBM24 and TARBP [75–79]. It is therefore conceivable that SRSF10 may associate with HBV nascent RNA in the nucleus to control its synthesis/elongation and/or its stability. Recent studies have indicated that HBV RNA stability is controlled by m-6A methylation [80,81]. SRSF10 may intervene in HBV RNA stability by interacting with m-6A readers or interfering with their RNA binding activity [82]. Future studies analyzing epigenetic changes and modifications in HBc/SRSF10 cccDNA/RNA binding activities in the presence of 1C8 should help uncover the underlying mechanisms.

Altogether, our study revealed that nuclear HBc can connect to an array of cellular RBPs. It also identified SRSF10 as a restriction factor in HBV viral RNA production, therefore providing a basis for the evaluation of a new class of host-targeted antiviral compounds that could improve the current anti-HBV arsenal and encourage combinational therapies.

## Methods

### Ethics statement

All animal experiments were performed in accordance with the European Union guidelines for approval of the protocols by the local ethics committee (Authorization Agreement C2EA-15, “Comité Rhône-Alpes d’Ethique pour l’Expérimentation Animale”, Lyon, France—APAFIS# 1570–2015073112163380 v4).

Primary human hepatocytes (PHH) were freshly prepared from human liver resections obtained with a formal written consent from the donors, at the Centre Léon Bérard (Lyon) with French ministerial authorizations (AC 2013–1871, DC 2013–1870, AFNOR NF 96 900 sept 2011).

### Cell culture and HBV infection

HepaRG cells were cultured, differentiated, and infected by HBV as previously described [34]. HepaRG-TR-HBc and HepaRG-TR-ST-HBc were obtained by transducing HepaRG-TR cells [83] with Lenti4/TO lentiviral vectors expressing either HBc or ST-HBc under the control of the minimal CMV/TetOn promoter. Details on sequences are available upon request. Transduced cells were selected using blasticin (10 μg/mL) and zeocyn (100 μg/mL), then amplified and frozen as polyclonal lines. Primary human hepatocytes (PHH) were freshly prepared from human liver resection as previously described [84]. HBV genotype D inoculum (subtype ayw) was prepared from HepAD38 [85] cell supernatant by polyethylene-glycol-MW-8000 (PEG8000, SIGMA) precipitation (8% final) as previously described [86]. Other HBV genotype viral inocula were similarly prepared from the supernatant of a newly developed stably-transformed HepG2 cell lines. Briefly, cell lines were obtained by transfection of a linearized pcDNA3Neo-HBV plasmid containing 1.35 genome unit of a consensus sequence of HBV genotype A, B, C, E, G, and H (obtained from HBV database: https://hbvdb.lyon.inserm.fr/; ref [87], the sequences are available upon request) and a double-round selection under G418 (500 ng/mL) by colony cell cloning (very low density seeding in large flasks). The titer of endotoxin free viral stocks was determined by qPCR. Cells were infected overnight in a media supplemented with 4% final of PEG, as previously described [34]. Infection was verified by measuring secreted HBsAg and HBeAg 7 to 10 days later by CLIA (Chemo-Luminescent Immune Assay) following manufacturer’s instructions (AutoBio, China).

### Production of AAV_HBV vectors

The AAV-HBVwt plasmid was obtained from ML Michel (Pasteur Institute) [88]. It contains the 1.2 genome copies of HBV genotype D sequence inserted between the AAV2 inverted terminal repeats (ITRs). The AAV-HBVnoHBc and AAV-HBVΔHBc vector plasmids were generated by inserting 1.3 genome units of a mutated HBV genotype D sequence between the AAV2 ITRs. The AAV-HBVnoHBc vector has a mutation in the HBc ATG (ATG to ATT). The AAV-HBVΔHBc has a deletion of the first 406 nucleotides of the HBc coding sequence. AAV serotype 3 vectors were produced by transient transfection of 293 cells and double purification on CsCl gradients by the Vector Core of the University of Nantes as previously described [89]. Vectors were titrated by qPCR using primers on the AAV ITRs.

### Chemical reagents

Unless otherwise specified, chemical reagents, drugs, antibiotics were purchased from Sigma Aldrich. Tenofovir (TDF) was a kind gift of Gilead Sciences (Foster city, USA). The core assembly modulator (CAM) used in the experiments was previously described [42], and resynthesized by AI-Biopharma (Montpellier, France). 1C8, *i*.*e*. 1C8, *i*.*e*. 1-Methyl-N-(5-nitrobenzo[d]isothiazol-3-yl)-4-oxo-1,4-dihydropyridine-3-carboxamide, was synthesized in Dr Gierson laboratory, but also re-synthesized and purified at 99% by AGV (Montpellier, France). RG7834 [90], a molecule that destabilizes HBV RNAs was synthetized by AI-Biopharma (Montpellier, France).

### Purification of ST-HBc complexes and sample preparation for mass spectrometry

HepaRG-TR-HBc and HepaRG-TR-ST-HBc cells (1,25x10^8^ cells) were first differentiated, then transgene expression induced for 72 hours by adding Tet (5 μg/ml) in the culture media. Nuclei were purified from cells using the NE-PER Nuclear and Cytoplasmic Extraction kit (Thermo Scientific), and nuclear extracts prepared by suspending nuclei in NER solution in the presence of protease inhibitors (Complete EDTA-free Protease Inhibitor Cocktail, Roche) and digestion or not with Benzonase (Sigma-Aldrich), during 40 min on ice. Nuclear extract recovered after centrifugation were added on Strep-Tactin gravity columns (IBA) and further purified following manufacturer’s instructions.

### Capsid migration assay

The intracellular formation of HBV nucleocapsids was assessed by native agarose gel electrophoresis of cell lysates, followed by transfer onto the enhanced chemiluminescence membrane and western blot analysis, as described previously [91].

### Mass spectrometry-based quantitative proteomic analyses

Eluted proteins were stacked in a single band in the top of a SDS-PAGE gel (4–12% NuPAGE, Life Technologies) and stained with Coomassie blue R-250 before in-gel digestion using modified trypsin (Promega, sequencing grade) as previously described [92]. Resulting peptides were analyzed by online nanoliquid chromatography coupled to tandem MS (UltiMate 3000 and LTQ-Orbitrap Velos Pro, Thermo Scientific). Peptides were sampled on a 300 μm x 5 mm PepMap C18 precolumn and separated on a 75 μm x 250 mm C18 column (PepMap, Thermo Scientific) using a 120-min gradient. MS and MS/MS data were acquired using Xcalibur (Thermo Scientific).

Peptides and proteins were identified and quantified using MaxQuant (version 1.5.3.30) [93] using the Uniprot database (*Homo sapiens* taxonomy proteome, October 2016 version), the sequence of HBc, and the frequently observed contaminant database embedded in MaxQuant. Trypsin was chosen as the enzyme and 2 missed cleavages were allowed. Peptide modifications allowed during the search were: carbamidomethylation (C, fixed), acetyl (Protein N-ter, variable) and oxidation (M, variable). Minimum peptide length was set to 7 amino acids. Minimum number of peptides, razor + unique peptides and unique peptides were set to 1. Maximum false discovery rates—calculated by employing a reverse database strategy—were set to 0.01 at PSM and protein levels. The “match between runs” option was activated. The iBAQ value [94] calculated by MaxQuant using razor + unique peptides was used to quantify proteins.

Statistical analysis were performed using ProStaR [95]. Proteins identified in the reverse and contaminant databases and proteins exhibiting less than 3 quantification values in one condition were discarded from the list. After log2 transformation, iBAQ values were normalized by median centering before missing value imputation (2.5-percentile value of each sample). Statistical testing was conducted using *limma* test. Differentially-expressed proteins were sorted out using a log_2_(fold change) cut-off of 2 and a p-value cut-off allowing to reach a FDR inferior to 1% according to the Benjamini-Hochberg procedure (p<0.0079 and p<0.0063 for dataset with and without Benzonase, respectively).

### Gene Ontology analyses and construction of the HBc-interactome

Proteomics data from the two conditions (without (benz-) or with (benz+) benzonase) were filtered out according to p-value (<0.005) and fold change (>4). Significant proteins common to the two conditions were extracted with a Venn diagram using their UniProtKB accession numbers. Statistical overrepresentation tests of these proteins were computed with PantherDB 11.1 and GO complete annotation sets. Overrepresented protein accession numbers were selected to further build and analyze their interacting network by means of Cytoscape software 3.5.1, querying IntAct molecular interaction database (May 27, 2017) with PSICQUIC service application 3.3.1, and Network Analyzer application 3.3.2.

### siRNA transfection

dHepaRG or PHH cells seeded into a 24-well plate were transfected with 25 nM or 10 nM of siRNA using Dharmafect#1 (GE HealthCare) or Lipofectamine RNAiMax (Life Technologies), respectively, following manufacturer’s instructions. SiRNA used were the following: siSRSF10 (Dharmacon SmartPool L-190401), siRBMX (Dharmacon SmartPool L-011691), siControl (Dharmacon D-001810).

### In vivo experiments

Primary human hepatocytes (PHH, Corning, BD Gentest or biopredic) were intrasplenically injected in NODFRG mice, a triple mutant mouse knocked-out for fumarylacetoacetate hydrolase (fah-/-), recombinase activating gene 2 (rag2-/-), interleukin 2 receptor gamma chain (IL2rg-/-). 48h after adeno-uPA conditioning [96], mice were subjected to NTBC (Swedish Orphan Biovitrum) cycling during the liver repopulation process, as described previously [97]. Mice with human serum albumin (HSA) levels >15 mg/mL, as determined using a Cobas C501 analyzer (Roche Applied Science), were inoculated with virus preparations by intra-peritoneal injection. Sera were collected at different time points before and after infection. Mice were sacrificed 10 weeks post-infection and liver sections snap frozen.

### Co-immunoprecipitation and western blot analysis

For Co-IP analyses, 300–500 μg of nuclear extracts, prepared as indicated above either from dHepaRG cells or from frozen liver sections were precleared with Protein A/G magnetic beads (Pierce) for 2hrs at 40°C and then incubated over-night at 4°C on a rotating wheel with 2 μg of anti HBc antibody (Dako B0586) or home generated anti-HBc (1/40000; generous gift from Dr Adam Zlotnick, Bloomington, USA). Immune-complexes were captured with protein A/G magnetic beads, washed four times in IP buffer and then eluted by boiling for 5 min in 2X loading buffer (Laemmli). For western blot, proteins were resolved by SDS-PAGE and then transferred onto a nitrocellulose membrane. Membranes were incubated with the primary antibodies corresponding to the indicated proteins. Proteins were revealed by chemi-luminescence (Super Signal West Dura Substrate, Pierce) using a secondary peroxydase-conjugated antibody (Dako) at a dilution of 1:10000. Primary antibodies used were: anti–HBc (Ab140243, 1/1000) or a home generated anti-HBc (1/40000; generous gift from Dr Adam Zlotnick, Bloomington, USA), anti-SRSF10 (Ab77209, 1/2000), anti-RBMX (Ab190352, 1/2000), anti-TRA2B (Ab171082, 1/2000), anti-SRSF1 (Ab38017, 1/1000), anti-SRSF2 (Ab204916, 1/1000), anti–DDX17 (Proteintech 19910-1-AP, 1/1000), anti-PARP1 (Ab6079, 1/1000), anti-DNAJB6 (Ab198995, 1/1000), anti-β-Tubulin (Ab6044, 1/10000), anti-NTCP (Ab131084, 1/1000), anti-Lamin B1 (Ab16048, 1/10000).

### Two-dimensional gel electrophoresis

For two-Dimensional (2D) gel electrophoresis 150 μg of nuclear proteins were dissolved in 150μl of 2D DeStreak Rehydration Solution and 0,5% IPG buffer (GE Healthcare). Samples were loaded on immobilized pH gradient Immobiline DryStrip gels pH 4–7 (GE Healthcare), isoelectrofocused with the Ettan IPGphor 3 Isoelectric Focusing System (GE Healthcare) according to the manufacturer’s instructions. The IPG strips were layered onto a 8–16% Criterion TGX Stain-Free Protein Gel (BIORAD). SDS-PAGE was performed with a Criterion cell (BIORAD) and blotted onto nitrocellulose membranes with Trans-Blot Turbo Transfer System (BIORAD) as recommended by the manufacturer. Membranes were then incubated with anti-SRSF10 antibody.

### Immunofluorescence analyses

Analyses were performed as described previously using anti-HBc (Thermo MA1-7607, 1/500) primary antibody and Alexa Fluor 555 secondary antibodies (Molecular Probes) [92]. Nuclei were stained with Hoescht 33258. Images were collected on a confocal NLO-LSM 880 microscope (Zeiss). Further image processing was performed using ICY [98].

### Nucleic acid extractions and analysis

Total RNA and DNA were extracted from cells with the NucleoSpin RNA II and Nucleospin 96 tissue kit, respectively, according to the manufacturer’s instructions (Macherey-Nagel). RNA reverse transcription was performed using SuperScript III (Invitrogen). Quantitative PCR for HBV were performed using HBV specific primers and normalized to PRNP housekeeping gene as previously described [99]. Pre-genomic RNA was quantified using the TaqMan Fast Advanced Master Mix (Life Technologies) and normalized to GusB cDNA levels. HBV cccDNA was quantified from total DNA following digestion for 45 min at 37°C with T5 exonuclease (Epicentre) to remove rcDNA followed by 30 min heat inactivation. cccDNA amount was quantified by TaqMan qPCR analyses and normalized to β-globin cDNA level, as previously described [100]. Analysis of HBV RNAs by Northern blot was performed as previously described [101]

### Analysis of spliced HBV RNA

The analysis of HBV spliced RNA was performed the RNomics platform of the University of Sherbrooke (Canada) as previously described [102,103]. After reverse-transcription, quantitative qPCR was performed using primers designed to detect each spliced and unspliced RNA and normalized to the MLRP19, PUM1 et YWHAZ genes (S2 Table). Primers were designed to detect 15 spliced RNA (sv1 to sv15) and 3 intronic regions (intron 1, 2 and 2b), as described in ref. [10].

### Quantification of nascent HBV RNA

HBV nascent RNA were quantified using the Click-iT Nascent RNA Capture Kit (Life Technologies) following the manufacturer’s instructions. Briefly HBV-infected dHepaRG were incubated for 2 hours with 5-ethynyl Uridine (EU) before RNA extraction and biotinylation. Control was provided by cells treated with Actinomycin D (1 μM) 20 min before labeling. Biotinylated RNA was purified on streptavidin magnetic beads. Total and EU-labeled RNA was reverse-transcribed and quantified as indicated above.

### Viability/cytotoxicity assays

Viability/cytotoxicity was assessed using the CellTiter-Glo Luminiscent assay (Promega) following the manufacturer’s instructions.

### Statistical analysis

Statistical analyses were performed using the XLStat software and Kruskal-Wallis tests with multiple comparison respect to non-treated cells (Dunn’s post-test). For all tests, a p value ≤ 0,05 was considered as significant. * correspond to p value ≤ 0.05; ** correspond to p value ≤ 0.01; *** correspond to p value ≤ 0.001.

## Supporting information

S1 Fig Functional analysis of dHepaRG-TR-ST-HBc cells. (A) Immunofluorescence (IF) of analysis of HBc localization in dHepaRG-TR-ST-HBc versus HBV-infected dHepaRG and PHH. (B) Intracellular HBV capsids, produced by the indicated cell lines, were analyzed by native gel electrophoresis followed by western blot with anti-HBc antibody. Lanes: 1. HepG2.2.15 2. dHepaRG-TR-HBe; 3. dHepaRG-TR-HBc; 4. dHepaRG-TR-ST-HBc.
(TIF)

S2 Fig Structure of SRSF10 isoforms detected in anti-HBc IP assays. (A) Structure of the two major SRSF10 isoforms. These two SRSF10 variants that migrate at 37 and 20–22 KDa correspond to the two major SRSF10 isoforms detected by the anti-SRSF10 antibody (Ab77209). Occasionally additional bands with an intermediate size are visible as shown in panel B. The red lines correspond to the regions targeted by the siRNA. (B) HBc was immune-precipitated from nuclear extracts of dHepaRG-HBc (HBc), dHepaRG-ST-HBc (ST-HBc) and control dHepaRG (RG) cells induced with Tet for two days. Eluted proteins were analyzed by western blot using anti-HBc and anti-SRSF10 antibodies. The asterisk indicates the positions of IgG heavy chain. (C) Putative SRSF10 isoform migrating between 25 and 35 KDa.
(TIF)

S3 Fig Effect of SRSF10 or RBMX KD on HBV replication in dHepaRG cells. (A) Outline of the experimental protocol in dHepaRG cells: cells were transfected with siRNA targeting SRSF10 or RBMX or control siRNA (siCTL) and then infected with HBV (MOI of 250 vge/cell). (B) NTCP levels in siRNA transfected dHepaRG cells before HBV infection (D0). **C.** Western blot validations in cells secreted parameters measured at D7pi. Results are expressed as the mean normalized ratio +/- SD, between siSRSF10 or siRBMX and siCTL transfected cells, of 3 independent experiments, each performed in triplicate.
(TIF)

S4 Fig Northern blot analysis. dHepaRG cells were transfected CTL or SRSF10 siRNA and infected with HBV as previously described (S3A Fig). Total RNA was extracted from cells at D7 pi and analyzed by Northern blot using HBV probes.
(TIF)

S5 Fig Effect of SRSF10 on established HBV replication. (A) Outline of the experimental protocol: dHepaRG cells were infected with HBV (MOI of 250 vge/cell) and then transfected twice with siRNA targeting SRSF10 or control siRNA (siCTL). Cells and supernatants were harvested at D15pi and analyzed to measure extracellular and intracellular HBV parameters. (B) Western blot validation of SRSF10 KD. (C) Effect of SRSF10 KD on intracellular and secreted HBV parameters. Results are expressed as the mean normalized ratio +/- SD, between siSRSF10 or siRBMX and siCTL transfected cells, of 3 independent experiments, each performed in triplicate.
(TIF)

S6 Fig Effect of SRSF10 KD on HBV RNAs produced in the absence of HBc. (A) dHepaRG cells were transfected with siRNA against SRSF10 or control siRNA and then transduced with AAV vectors containing either a wt (AAVHBVwt) or an HBc-deficient genome (AAVHBVnoHBc and AAVHBVΔHBc) at a MOI of 10^4^ vge/cell. Secreted antigens and total RNAs were quantified 10 days later. Results are expressed as the mean normalized ratio +/- SD, between siSRSF10 and siCTL transfected cells, of 3 independent experiments, each performed in triplicate.
(TIF)

S7 Fig Analysis of SRSF10 phosphorylation by 2D-gel electrophoresis. Nuclear extracts were prepared from dHepaRG cells either mock (A) or 1C8-treated (18 hrs at 20μM) (B) and separated by two-dimensional gel electrophoresis followed by western blot using an anti-SRSF10 antibody. Numbers on the top of the images indicate the pH gradient. Only the larger SRSF10 isoform of 37 KDa was visible under these conditions. The arrow indicates a hypo-phosphorylated isoform generated following 1C8 treatment.
(TIF)

S8 Fig Characterization of 1C8 EC50 and toxicity assay. (A) to (D). Measure of 1C8 EC50 on HBV-infected dHepaRG. dHepaRG cells were infected with HBV (MOI of 250 vge/cell) for 7 days followed by three treatments with increasing concentration of 1C8. Total HBV RNAs (A), secreted HBV DNA (B), HBsAg (C) and HBeAg (D) were measured two days after the last treatment. Results are presented as the mean change in expression or secretion +/- SD of three independent experiments, each performed in triplicate. (E) Toxicity assay. Cell viability of dHepaRG cells treated with increasing concentrations of 1C8, was measured using the CellTiter-Glo Luminiscent Cell Viability Assay (Promega). Non-infected (NI) and HBV-infected dHepaRG cells treated with DMSO and puromycin were used as negative and positive controls, respectively.
(TIF)

S9 Fig Effect of 1C8 on the replication of various HBV genotypes in dHepaRG cells. (A) Cells were infected with HBV genotype C (MOI of 100 vge/cell) and treated as indicated in Fig 5A. Treatments included, Tenofovir (TDF at 10 μM), a Core allosteric modulator (CAM at 10 μM) or 1C8 (10 μM). Intracellular and secreted HBV parameters were quantified 2 days after the last treatment. Results are expressed as the mean normalized ratio +/- SD between non-treated and treated cells of 2 independent experiments, each performed in triplicate. (B) Cells were infected with indicated HBV genotypes (MOI of 100 vge/cell) and either mock- or treated with 1C8 (10 μM). HBeAg and HBsAg were quantified by CLIA. Results are expressed as the mean normalized ratio +/- SD between non-treated and treated cells of a single experiment, with biological triplicates.
(TIF)

S10 Fig Analysis of spliced and unspliced HBV RNA following SRSF10 KD or 1C8 treatment. (A) and (B) Total RNA were extracted from dHepaRG (A) and PHH (B) transfected with siRNA following the previously described protocol (Fig 4A). (C) HBV-infected dHepaRG were treated with 1C8 as previously described (Fig 5B). HBV RNAs were analyzed by end-point RT-qPCR using sets of primers able to discriminate each spliced and unspliced form (see Methods section). Results are expressed as the mean ratio +/- SD between siSRSF10 and siCTL transfected cells of 3(A and C) or 2 (B) independent experiments.
(TIF)

S1 Table HBc interactome analysis using MS-based quantitative proteomics. ST-HBc-associated proteins were identified using MS-based proteomics. For this, cell lysate expressing ST-HBc or untagged HBc were treated or not with Benzonase before purification by affinity using Strep-Tactin. Eluted proteins were digested with trypsin and the resulting peptides submitted to MS-based proteomic analysis. The proteins were then identified and quantified in each sample before statistical analysis allowing to sort out proteins enriched with ST-HBc compared to untagged HBc used as negative control.
(XLSX)

S2 Table List of primers used for the quantification of spliced and unspliced HBV RNAs. (XLSX)

## References

[1] Levrero M, Zucman-Rossi J. Mechanisms of HBV-induced hepatocellular carcinoma. J Hepatol. 2016;64(1 Suppl):S84–101. 10.1016/j.jhep.2016.02.021 . 27084040

[2] Zoulim F, Durantel D. Antiviral therapies and prospects for a cure of chronic hepatitis B. Cold Spring Harb Perspect Med. 2015;5(4). 10.1101/cshperspect.a021501 . 25833942 PMC4382723

[3] Zeisel MB, Lucifora J, Mason WS, Sureau C, Beck J, Levrero M, et al Towards an HBV cure: state-of-the-art and unresolved questions—report of the ANRS workshop on HBV cure. Gut. 2015;64(8):1314–26. 10.1136/gutjnl-2014-308943 . 25670809

[4] Durantel D, Zoulim F. New antiviral targets for innovative treatment concepts for hepatitis B virus and hepatitis delta virus. J Hepatol. 2016;64(1 Suppl):S117–31. 10.1016/j.jhep.2016.02.016 . 27084032

[5] Fanning GC, Zoulim F, Hou J, Bertoletti A. Therapeutic strategies for hepatitis B virus infection: towards a cure. Nat Rev Drug Discov. 2019 Epub 2019/08/29. 10.1038/s41573-019-0037-0 . 31455905

[6] Seeger C, Mason WS. Molecular biology of hepatitis B virus infection. Virology. 2015;479–480:672–86. 10.1016/j.virol.2015.02.031 25759099 PMC4424072

[7] Koniger C, Wingert I, Marsmann M, Rosler C, Beck J, Nassal M. Involvement of the host DNA-repair enzyme TDP2 in formation of the covalently closed circular DNA persistence reservoir of hepatitis B viruses. Proc Natl Acad Sci U S A. 2014;111(40):E4244–53. Epub 2014/09/10. 10.1073/pnas.1409986111 25201958 PMC4209993

[8] Nassal M. HBV cccDNA: viral persistence reservoir and key obstacle for a cure of chronic hepatitis B. Gut. 2015;64(12):1972–84. 10.1136/gutjnl-2015-309809 . 26048673

[9] Wei L, Ploss A. Core components of DNA lagging strand synthesis machinery are essential for hepatitis B virus cccDNA formation. Nat Microbiol. 2020 Epub 2020/03/11. 10.1038/s41564-020-0678-0 . 32152586 PMC7190442

[10] Sommer G, Heise T. Posttranscriptional control of HBV gene expression. Front Biosci. 2008;13:5533–47. PubMed 10.2741/3097 . 18508603

[11] Redelsperger F, Lekbaby B, Mandouri Y, Giang E, Duriez M, Desire N, et al Production of hepatitis B defective particles is dependent on liver status. Virology. 2012;431(1–2):21–8. Epub 2012/06/06. 10.1016/j.virol.2012.05.008 . 22664356

[12] Zlotnick A, Venkatakrishnan B, Tan Z, Lewellyn E, Turner W, Francis S. Core protein: A pleiotropic keystone in the HBV lifecycle. Antiviral Res. 2015;121:82–93. 10.1016/j.antiviral.2015.06.020 26129969 PMC4537649

[13] Chu TH, Liou AT, Su PY, Wu HN, Shih C. Nucleic acid chaperone activity associated with the arginine-rich domain of human hepatitis B virus core protein. J Virol. 2014;88(5):2530–43. Epub 2013/12/20. 10.1128/JVI.03235-13 24352445 PMC3958103

[14] Li HC, Huang EY, Su PY, Wu SY, Yang CC, Lin YS, et al Nuclear export and import of human hepatitis B virus capsid protein and particles. PLoS Pathog. 2010;6(10):e1001162 Epub 2010/11/10. 10.1371/journal.ppat.1001162 21060813 PMC2965763

[15] Liu K, Ludgate L, Yuan Z, Hu J. Regulation of multiple stages of hepadnavirus replication by the carboxyl-terminal domain of viral core protein in trans. J Virol. 2015;89(5):2918–30. Epub 2014/12/30. 10.1128/JVI.03116-14 25540387 PMC4325754

[16] Nassal M. The arginine-rich domain of the hepatitis B virus core protein is required for pregenome encapsidation and productive viral positive-strand DNA synthesis but not for virus assembly. J Virol. 1992;66(7):4107–16. Epub 1992/07/01. PubMed 10.1128/JVI.66.7.4107-4116.1992 1602535 PMC241213

[17] Diab A, Foca A, Zoulim F, Durantel D, Andrisani O. The diverse functions of the hepatitis B core/capsid protein (HBc) in the viral life cycle: Implications for the development of HBc-targeting antivirals. Antiviral Res. 2018;149:211–20. 10.1016/j.antiviral.2017.11.015 29183719 PMC5757518

[18] Rabe B, Delaleau M, Bischof A, Foss M, Sominskaya I, Pumpens P, et al Nuclear entry of hepatitis B virus capsids involves disintegration to protein dimers followed by nuclear reassociation to capsids. PLoS Pathog. 2009;5(8):e1000563 10.1371/journal.ppat.1000563 19714236 PMC2727048

[19] Blondot ML, Bruss V, Kann M. Intracellular transport and egress of hepatitis B virus. J Hepatol. 2016;64(1 Suppl):S49–59. 10.1016/j.jhep.2016.02.008 . 27084037

[20] Akiba T, Nakayama H, Miyazaki Y, Kanno A, Ishii M, Ohori H. Relationship between the replication of hepatitis B virus and the localization of virus nucleocapsid antigen (HBcAg) in hepatocytes. J Gen Virol. 1987;68 (Pt 3):871–7. Epub 1987/03/01. 10.1099/0022-1317-68-3-871 . 3819701

[21] Deroubaix A, Osseman Q, Cassany A, Begu D, Ragues J, Kassab S, et al Expression of viral polymerase and phosphorylation of core protein determine core and capsid localization of the human hepatitis B virus. J Gen Virol. 2015;96(Pt 1):183–95. Epub 2014/10/03. 10.1099/vir.0.064816-0 . 25274856

[22] Guidotti LG, Martinez V, Loh YT, Rogler CE, Chisari FV. Hepatitis B virus nucleocapsid particles do not cross the hepatocyte nuclear membrane in transgenic mice. J Virol. 1994;68(9):5469–75. Epub 1994/09/01. PubMed 10.1128/JVI.68.9.5469-5475.1994 8057429 PMC236947

[23] Zhang X, Lu W, Zheng Y, Wang W, Bai L, Chen L, et al In situ analysis of intrahepatic virological events in chronic hepatitis B virus infection. J Clin Invest. 2016;126(3):1079–92. 10.1172/JCI83339 26901811 PMC4767362

[24] Bock CT, Schwinn S, Locarnini S, Fyfe J, Manns MP, Trautwein C, et al Structural organization of the hepatitis B virus minichromosome. J Mol Biol. 2001;307(1):183–96. 10.1006/jmbi.2000.4481 . 11243813

[25] Hatton T, Zhou S, Standring DN. RNA- and DNA-binding activities in hepatitis B virus capsid protein: a model for their roles in viral replication. J Virol. 1992;66(9):5232–41. PubMed 10.1128/JVI.66.9.5232-5241.1992 1501273 PMC289076

[26] Pollicino T, Belloni L, Raffa G, Pediconi N, Squadrito G, Raimondo G, et al Hepatitis B virus replication is regulated by the acetylation status of hepatitis B virus cccDNA-bound H3 and H4 histones. Gastroenterology. 2006;130(3):823–37. Epub 2006/03/15. 10.1053/j.gastro.2006.01.001 16530522

[27] Guo YH, Li YN, Zhao JR, Zhang J, Yan Z. HBc binds to the CpG islands of HBV cccDNA and promotes an epigenetic permissive state. Epigenetics. 2011;6(6):720–6. Epub 2011/05/07. 10.4161/epi.6.6.15815 . 21546797

[28] Chong CK, Cheng CYS, Tsoi SYJ, Huang FY, Liu F, Seto WK, et al Role of hepatitis B core protein in HBV transcription and recruitment of histone acetyltransferases to cccDNA minichromosome. Antiviral Res. 2017;144:1–7. Epub 2017/05/14. 10.1016/j.antiviral.2017.05.003 . 28499864

[29] Guo Y, Kang W, Lei X, Li Y, Xiang A, Liu Y, et al Hepatitis B viral core protein disrupts human host gene expression by binding to promoter regions. BMC Genomics. 2012;13:563 10.1186/1471-2164-13-563 23088787 PMC3484065

[30] McGonigle R, Yap WB, Ong ST, Gatherer D, Bakker SE, Tan WS, et al An N-terminal extension to the hepatitis B virus core protein forms a poorly ordered trimeric spike in assembled virus-like particles. Journal of structural biology. 2015;189(2):73–80. Epub 2015/01/06. 10.1016/j.jsb.2014.12.006 25557498 PMC4318616

[31] Long JC, Caceres JF. The SR protein family of splicing factors: master regulators of gene expression. Biochem J. 2009;417(1):15–27. 10.1042/BJ20081501 . 19061484

[32] Jeong S. SR Proteins: Binders, Regulators, and Connectors of RNA. Mol Cells. 2017;40(1):1–9. Epub 2017/02/06. 10.14348/molcells.2017.2319 28152302 PMC5303883

[33] Twyffels L, Gueydan C, Kruys V. Shuttling SR proteins: more than splicing factors. FEBS J. 2011;278(18):3246–55. Epub 2011/07/29. 10.1111/j.1742-4658.2011.08274.x . 21794093

[34] Gripon P, Rumin S, Urban S, Le Seyec J, Glaise D, Cannie I, et al Infection of a human hepatoma cell line by hepatitis B virus. Proc Natl Acad Sci U S A. 2002;99(24):15655–60. Epub 2002/11/15. 10.1073/pnas.232137699 12432097 PMC137772

[35] Hantz O, Parent R, Durantel D, Gripon P, Guguen-Guillouzo C, Zoulim F. Persistence of the hepatitis B virus covalently closed circular DNA in HepaRG human hepatocyte-like cells. J Gen Virol. 2009;90(Pt 1):127–35. 10.1099/vir.0.004861-0 . 19088281

[36] Shin C, Manley JL. The SR protein SRp38 represses splicing in M phase cells. Cell. 2002;111(3):407–17. PubMed 10.1016/s0092-8674(02)01038-3 . 12419250

[37] Feng Y, Chen M, Manley JL. Phosphorylation switches the general splicing repressor SRp38 to a sequence-specific activator. Nat Struct Mol Biol. 2008;15(10):1040–8. Epub 2008/09/17. 10.1038/nsmb.1485 18794844 PMC2668916

[38] Shi Y, Manley JL. A complex signaling pathway regulates SRp38 phosphorylation and pre-mRNA splicing in response to heat shock. Mol Cell. 2007;28(1):79–90. 10.1016/j.molcel.2007.08.028 . 17936706

[39] Shin C, Feng Y, Manley JL. Dephosphorylated SRp38 acts as a splicing repressor in response to heat shock. Nature. 2004;427(6974):553–8. 10.1038/nature02288 . 14765198

[40] Cheung PK, Horhant D, Bandy LE, Zamiri M, Rabea SM, Karagiosov SK, et al A Parallel Synthesis Approach to the Identification of Novel Diheteroarylamide-Based Compounds Blocking HIV Replication: Potential Inhibitors of HIV-1 Pre-mRNA Alternative Splicing. J Med Chem. 2016;59(5):1869–79. Epub 2016/02/16. 10.1021/acs.jmedchem.5b01357 . 26878150

[41] Shkreta L, Blanchette M, Toutant J, Wilhelm E, Bell B, Story BA, et al Modulation of the splicing regulatory function of SRSF10 by a novel compound that impairs HIV-1 replication. Nucleic Acids Res. 2017;45(7):4051–67. 10.1093/nar/gkw1223 27928057 PMC5397194

[42] Lahlali T, Berke JM, Vergauwen K, Foca A, Vandyck K, Pauwels F, et al Novel Potent Capsid Assembly Modulators Regulate Multiple Steps of the Hepatitis B Virus Life Cycle. Antimicrob Agents Chemother. 2018;62(10). Epub 2018/07/18. 10.1128/AAC.00835-18 30012770 PMC6153789

[43] Huang HL, Jeng KS, Hu CP, Tsai CH, Lo SJ, Chang C. Identification and characterization of a structural protein of hepatitis B virus: a polymerase and surface fusion protein encoded by a spliced RNA. Virology. 2000;275(2):398–410. Epub 2000/09/22. 10.1006/viro.2000.0478 . 10998339

[44] Soussan P, Garreau F, Zylberberg H, Ferray C, Brechot C, Kremsdorf D. In vivo expression of a new hepatitis B virus protein encoded by a spliced RNA. J Clin Invest. 2000;105(1):55–60. Epub 2000/01/05. 10.1172/JCI8098 10619861 PMC382588

[45] Marion MJ, Hantz O, Durantel D. The HepaRG cell line: biological properties and relevance as a tool for cell biology, drug metabolism, and virology studies. Methods Mol Biol. 2010;640:261–72. 10.1007/978-1-60761-688-7_13 . 20645056

[46] Hirose T, Nakagawa S. Paraspeckles: possible nuclear hubs by the RNA for the RNA. Biomol Concepts. 2012;3(5):415–28. Epub 2012/10/01. 10.1515/bmc-2012-0017 . 25436547

[47] Spector DL, Lamond AI. Nuclear speckles. Cold Spring Harb Perspect Biol. 2011;3(2). Epub 2010/10/12. 10.1101/cshperspect.a000646 20926517 PMC3039535

[48] Chen Y, Zhang Y, Wang Y, Zhang L, Brinkman EK, Adam SA, et al Mapping 3D genome organization relative to nuclear compartments using TSA-Seq as a cytological ruler. J Cell Biol. 2018;217(11):4025–48. Epub 2018/08/30. 10.1083/jcb.201807108 30154186 PMC6219710

[49] Kim J, Venkata NC, Hernandez Gonzalez GA, Khanna N, Belmont AS. Gene expression amplification by nuclear speckle association. J Cell Biol. 2020;219(1). Epub 2019/11/24. 10.1083/jcb.201904046 . 31757787 PMC7039209

[50] Hentze MW, Castello A, Schwarzl T, Preiss T. A brave new world of RNA-binding proteins. Nat Rev Mol Cell Biol. 2018;19(5):327–41. Epub 2018/01/18. 10.1038/nrm.2017.130 . 29339797

[51] Ricco R, Kanduc D. Hepatitis B virus and Homo sapiens proteome-wide analysis: A profusion of viral peptide overlaps in neuron-specific human proteins. Biologics. 2010;4:75–81. PubMed 10.2147/btt.s8890 20531967 PMC2880343

[52] Corley M, Burns MC, Yeo GW. How RNA-Binding Proteins Interact with RNA: Molecules and Mechanisms. Mol Cell. 2020;78(1):9–29. Epub 2020/04/04. 10.1016/j.molcel.2020.03.011 . 32243832 PMC7202378

[53] Heger-Stevic J, Zimmermann P, Lecoq L, Bottcher B, Nassal M. Hepatitis B virus core protein phosphorylation: Identification of the SRPK1 target sites and impact of their occupancy on RNA binding and capsid structure. PLoS Pathog. 2018;14(12):e1007488 10.1371/journal.ppat.1007488 30566530 PMC6317823

[54] Conrad T, Albrecht AS, de Melo Costa VR, Sauer S, Meierhofer D, Orom UA. Serial interactome capture of the human cell nucleus. Nat Commun. 2016;7:11212 Epub 2016/04/05. 10.1038/ncomms11212 27040163 PMC4822031

[55] Adamson B, Smogorzewska A, Sigoillot FD, King RW, Elledge SJ. A genome-wide homologous recombination screen identifies the RNA-binding protein RBMX as a component of the DNA-damage response. Nat Cell Biol. 2012;14(3):318–28. 10.1038/ncb2426 22344029 PMC3290715

[56] Matsunaga S, Takata H, Morimoto A, Hayashihara K, Higashi T, Akatsuchi K, et al RBMX: a regulator for maintenance and centromeric protection of sister chromatid cohesion. Cell Rep. 2012;1(4):299–308. 10.1016/j.celrep.2012.02.005 . 22832223

[57] Shin C, Kleiman FE, Manley JL. Multiple properties of the splicing repressor SRp38 distinguish it from typical SR proteins. Mol Cell Biol. 2005;25(18):8334–43. 10.1128/MCB.25.18.8334-8343.2005 16135820 PMC1234314

[58] Shkreta L, Toutant J, Durand M, Manley JL, Chabot B. SRSF10 Connects DNA Damage to the Alternative Splicing of Transcripts Encoding Apoptosis, Cell-Cycle Control, and DNA Repair Factors. Cell Rep. 2016;17(8):1990–2003. 10.1016/j.celrep.2016.10.071 27851963 PMC5483951

[59] Zhou X, Li X, Cheng Y, Wu W, Xie Z, Xi Q, et al BCLAF1 and its splicing regulator SRSF10 regulate the tumorigenic potential of colon cancer cells. Nat Commun. 2014;5:4581 10.1038/ncomms5581 . 25091051

[60] Zhou X, Wu W, Li H, Cheng Y, Wei N, Zong J, et al Transcriptome analysis of alternative splicing events regulated by SRSF10 reveals position-dependent splicing modulation. Nucleic Acids Res. 2014;42(6):4019–30. 10.1093/nar/gkt1387 24442672 PMC3973337

[61] Hennig S, Kong G, Mannen T, Sadowska A, Kobelke S, Blythe A, et al Prion-like domains in RNA binding proteins are essential for building subnuclear paraspeckles. J Cell Biol. 2015;210(4):529–39. Epub 2015/08/19. 10.1083/jcb.201504117 26283796 PMC4539981

[62] An H, Tan JT, Shelkovnikova TA. Stress granules regulate stress-induced paraspeckle assembly. J Cell Biol. 2019;218(12):4127–40. Epub 2019/10/23. 10.1083/jcb.201904098 31636118 PMC6891081

[63] Ninomiya K, Adachi S, Natsume T, Iwakiri J, Terai G, Asai K, et al LncRNA-dependent nuclear stress bodies promote intron retention through SR protein phosphorylation. EMBO J. 2020;39(3):e102729 Epub 2019/11/30. 10.15252/embj.2019102729 31782550 PMC6996502

[64] Brillen AL, Walotka L, Hillebrand F, Muller L, Widera M, Theiss S, et al Analysis of Competing HIV-1 Splice Donor Sites Uncovers a Tight Cluster of Splicing Regulatory Elements within Exon 2/2b. J Virol. 2017;91(14). Epub 2017/04/28. 10.1128/JVI.00389-17 28446664 PMC5487545

[65] Ludgate L, Liu K, Luckenbaugh L, Streck N, Eng S, Voitenleitner C, et al Cell-Free Hepatitis B Virus Capsid Assembly Dependent on the Core Protein C-Terminal Domain and Regulated by Phosphorylation. J Virol. 2016;90(12):5830–44. Epub 2016/04/15. 10.1128/JVI.00394-16 27076641 PMC4886785

[66] Zhao Q, Hu Z, Cheng J, Wu S, Luo Y, Chang J, et al Hepatitis B Virus Core Protein Dephosphorylation Occurs during Pregenomic RNA Encapsidation. J Virol. 2018;92(13). Epub 2018/04/20. 10.1128/JVI.02139-17 29669831 PMC6002726

[67] Daub H, Blencke S, Habenberger P, Kurtenbach A, Dennenmoser J, Wissing J, et al Identification of SRPK1 and SRPK2 as the major cellular protein kinases phosphorylating hepatitis B virus core protein. J Virol. 2002;76(16):8124–37. PubMed 10.1128/jvi.76.16.8124-8137.2002 12134018 PMC155132

[68] Gazina EV, Fielding JE, Lin B, Anderson DA. Core protein phosphorylation modulates pregenomic RNA encapsidation to different extents in human and duck hepatitis B viruses. J Virol. 2000;74(10):4721–8. Epub 2000/04/25. 10.1128/jvi.74.10.4721-4728.2000 10775610 PMC111994

[69] Perlman DH, Berg EA, O'Connor P B, Costello CE, Hu J. Reverse transcription-associated dephosphorylation of hepadnavirus nucleocapsids. Proc Natl Acad Sci U S A. 2005;102(25):9020–5. 10.1073/pnas.0502138102 15951426 PMC1157036

[70] Ye F, Chen ER, Nilsen TW. Kaposi's Sarcoma-Associated Herpesvirus Utilizes and Manipulates RNA N(6)-Adenosine Methylation To Promote Lytic Replication. J Virol. 2017;91(16). Epub 2017/06/09. 10.1128/JVI.00466-17 28592530 PMC5533915

[71] Mahiet C, Swanson CM. Control of HIV-1 gene expression by SR proteins. Biochem Soc Trans. 2016;44(5):1417–25. Epub 2016/12/03. 10.1042/BST20160113 . 27911724

[72] Ji X, Zhou Y, Pandit S, Huang J, Li H, Lin CY, et al SR proteins collaborate with 7SK and promoter-associated nascent RNA to release paused polymerase. Cell. 2013;153(4):855–68. Epub 2013/05/15. 10.1016/j.cell.2013.04.028 23663783 PMC4103662

[73] Lin S, Coutinho-Mansfield G, Wang D, Pandit S, Fu XD. The splicing factor SC35 has an active role in transcriptional elongation. Nat Struct Mol Biol. 2008;15(8):819–26. Epub 2008/07/22. 10.1038/nsmb.1461 18641664 PMC2574591

[74] Zhong XY, Wang P, Han J, Rosenfeld MG, Fu XD. SR proteins in vertical integration of gene expression from transcription to RNA processing to translation. Mol Cell. 2009;35(1):1–10. Epub 2009/07/15. 10.1016/j.molcel.2009.06.016 19595711 PMC2744344

[75] Makokha GN, Abe-Chayama H, Chowdhury S, Hayes CN, Tsuge M, Yoshima T, et al Regulation of the Hepatitis B virus replication and gene expression by the multi-functional protein TARDBP. Sci Rep. 2019;9(1):8462 Epub 2019/06/13. 10.1038/s41598-019-44934-5 31186504 PMC6560085

[76] Ng LF, Chan M, Chan SH, Cheng PC, Leung EH, Chen WN, et al Host heterogeneous ribonucleoprotein K (hnRNP K) as a potential target to suppress hepatitis B virus replication. PLoS Med. 2005;2(7):e163 Epub 2005/07/22. 10.1371/journal.pmed.0020163 16033304 PMC1181871

[77] Sun S, Nakashima K, Ito M, Li Y, Chida T, Takahashi H, et al Involvement of PUF60 in Transcriptional and Post-transcriptional Regulation of Hepatitis B Virus Pregenomic RNA Expression. Sci Rep. 2017;7(1):12874 Epub 2017/10/11. 10.1038/s41598-017-12497-y 28993636 PMC5634508

[78] Tay N, Chan SH, Ren EC. Identification and cloning of a novel heterogeneous nuclear ribonucleoprotein C-like protein that functions as a transcriptional activator of the hepatitis B virus enhancer II. J Virol. 1992;66(12):6841–8. Epub 1992/12/01. PubMed 10.1128/JVI.66.12.6841-6848.1992 1433497 PMC240284

[79] Yao Y, Yang B, Cao H, Zhao K, Yuan Y, Chen Y, et al RBM24 stabilizes hepatitis B virus pregenomic RNA but inhibits core protein translation by targeting the terminal redundancy sequence. Emerg Microbes Infect. 2018;7(1):86 Epub 2018/05/16. 10.1038/s41426-018-0091-4 29760415 PMC5951808

[80] Imam H, Khan M, Gokhale NS, McIntyre ABR, Kim GW, Jang JY, et al N6-methyladenosine modification of hepatitis B virus RNA differentially regulates the viral life cycle. Proc Natl Acad Sci U S A. 2018;115(35):8829–34. Epub 2018/08/15. 10.1073/pnas.1808319115 30104368 PMC6126736

[81] Imam H, Kim GW, Mir SA, Khan M, Siddiqui A. Interferon-stimulated gene 20 (ISG20) selectively degrades N6-methyladenosine modified Hepatitis B Virus transcripts. PLoS Pathog. 2020;16(2):e1008338 Epub 2020/02/15. 10.1371/journal.ppat.1008338 32059034 PMC7046284

[82] Xiao W, Adhikari S, Dahal U, Chen YS, Hao YJ, Sun BF, et al Nuclear m(6)A Reader YTHDC1 Regulates mRNA Splicing. Mol Cell. 2016;61(4):507–19. Epub 2016/02/16. 10.1016/j.molcel.2016.01.012 . 26876937

[83] Lucifora J, Arzberger S, Durantel D, Belloni L, Strubin M, Levrero M, et al Hepatitis B virus X protein is essential to initiate and maintain virus replication after infection. J Hepatol. 2011;55(5):996–1003. Epub 2011/03/08. 10.1016/j.jhep.2011.02.015 . 21376091

[84] Lecluyse EL, Alexandre E. Isolation and culture of primary hepatocytes from resected human liver tissue. Methods Mol Biol. 2010;640:57–82. Epub 2010/07/21. 10.1007/978-1-60761-688-7_3 . 20645046

[85] Ladner SK, Otto MJ, Barker CS, Zaifert K, Wang GH, Guo JT, et al Inducible expression of human hepatitis B virus (HBV) in stably transfected hepatoblastoma cells: a novel system for screening potential inhibitors of HBV replication. Antimicrob Agents Chemother. 1997;41(8):1715–20. Epub 1997/08/01. PubMed 10.1128/AAC.41.8.1715 9257747 PMC163991

[86] Luangsay S, Gruffaz M, Isorce N, Testoni B, Michelet M, Faure-Dupuy S, et al Early inhibition of hepatocyte innate responses by hepatitis B virus. J Hepatol. 2015;63(6):1314–22. 10.1016/j.jhep.2015.07.014 . 26216533

[87] Hayer J, Jadeau F, Deleage G, Kay A, Zoulim F, Combet C. HBVdb: a knowledge database for Hepatitis B Virus. Nucleic Acids Res. 2013;41(Database issue):D566–70. Epub 2012/11/06. 10.1093/nar/gks1022 23125365 PMC3531116

[88] Dion S, Bourgine M, Godon O, Levillayer F, Michel ML. Adeno-associated virus-mediated gene transfer leads to persistent hepatitis B virus replication in mice expressing HLA-A2 and HLA-DR1 molecules. J Virol. 2013;87(10):5554–63. 10.1128/JVI.03134-12 23468504 PMC3648192

[89] Salvetti A, Oreve S, Chadeuf G, Favre D, Cherel Y, Champion-Arnaud P, et al Factors influencing recombinant adeno-associated virus production. Human Gene Therapy. 1998;9(5):695–706. 10.1089/hum.1998.9.5-695 WOS:000072839500010. 9551617

[90] Mueller H, Wildum S, Luangsay S, Walther J, Lopez A, Tropberger P, et al A novel orally available small molecule that inhibits hepatitis B virus expression. J Hepatol. 2018;68(3):412–20. Epub 2017/10/29. 10.1016/j.jhep.2017.10.014 . 29079285

[91] Ning X, Basagoudanavar SH, Liu K, Luckenbaugh L, Wei D, Wang C, et al Capsid Phosphorylation State and Hepadnavirus Virion Secretion. J Virol. 2017;91(9). 10.1128/JVI.00092-17 28228589 PMC5391479

[92] Salvetti A, Coute Y, Epstein A, Arata L, Kraut A, Navratil V, et al Nuclear Functions of Nucleolin through Global Proteomics and Interactomic Approaches. J Proteome Res. 2016;15(5):1659–69. 10.1021/acs.jproteome.6b00126 . 27049334

[93] Tyanova S, Temu T, Cox J. The MaxQuant computational platform for mass spectrometry-based shotgun proteomics. Nat Protoc. 2016;11(12):2301–19. Epub 2016/11/04. 10.1038/nprot.2016.136 . 27809316

[94] Schwanhausser B, Busse D, Li N, Dittmar G, Schuchhardt J, Wolf J, et al Global quantification of mammalian gene expression control. Nature. 2011;473(7347):337–42. Epub 2011/05/20. 10.1038/nature10098 . 21593866

[95] Wieczorek S, Combes F, Lazar C, Giai Gianetto Q, Gatto L, Dorffer A, et al DAPAR & ProStaR: software to perform statistical analyses in quantitative discovery proteomics. Bioinformatics. 2017;33(1):135–6. 10.1093/bioinformatics/btw580 27605098 PMC5408771

[96] Bissig KD, Le TT, Woods NB, Verma IM. Repopulation of adult and neonatal mice with human hepatocytes: a chimeric animal model. Proc Natl Acad Sci U S A. 2007;104(51):20507–11. Epub 2007/12/14. 10.1073/pnas.0710528105 18077355 PMC2154461

[97] Calattini S, Fusil F, Mancip J, Dao Thi VL, Granier C, Gadot N, et al Functional and Biochemical Characterization of Hepatitis C Virus (HCV) Particles Produced in a Humanized Liver Mouse Model. The Journal of biological chemistry. 2015;290(38):23173–87. Epub 2015/08/01. 10.1074/jbc.M115.662999 26224633 PMC4645586

[98] de Chaumont F, Dallongeville S, Chenouard N, Herve N, Pop S, Provoost T, et al Icy: an open bioimage informatics platform for extended reproducible research. Nat Methods. 2012;9(7):690–6. Epub 2012/06/30. 10.1038/nmeth.2075 . 22743774

[99] Lucifora J, Xia Y, Reisinger F, Zhang K, Stadler D, Cheng X, et al Specific and nonhepatotoxic degradation of nuclear hepatitis B virus cccDNA. Science. 2014;343(6176):1221–8. 10.1126/science.1243462 . 24557838 PMC6309542

[100] Werle-Lapostolle B, Bowden S, Locarnini S, Wursthorn K, Petersen J, Lau G, et al Persistence of cccDNA during the natural history of chronic hepatitis B and decline during adefovir dipivoxil therapy. Gastroenterology. 2004;126(7):1750–8. Epub 2004/06/10. 10.1053/j.gastro.2004.03.018 . 15188170

[101] Alfaiate D, Lucifora J, Abeywickrama-Samarakoon N, Michelet M, Testoni B, Cortay JC, et al HDV RNA replication is associated with HBV repression and interferon-stimulated genes induction in super-infected hepatocytes. Antiviral Res. 2016;136:19–31. 10.1016/j.antiviral.2016.10.006 . 27771387

[102] Brosseau JP, Lucier JF, Lapointe E, Durand M, Gendron D, Gervais-Bird J, et al High-throughput quantification of splicing isoforms. RNA. 2010;16(2):442–9. Epub 2009/12/30. 10.1261/rna.1877010 20038630 PMC2811672

[103] Prinos P, Garneau D, Lucier JF, Gendron D, Couture S, Boivin M, et al Alternative splicing of SYK regulates mitosis and cell survival. Nat Struct Mol Biol. 2011;18(6):673–9. Epub 2011/05/10. 10.1038/nsmb.2040 . 21552259

[104] Smits AH, Jansen PW, Poser I, Hyman AA, Vermeulen M. Stoichiometry of chromatin-associated protein complexes revealed by label-free quantitative mass spectrometry-based proteomics. Nucleic Acids Res. 2013;41(1):e28 Epub 2012/10/16. 10.1093/nar/gks941 23066101 PMC3592467

