# Supplementary figures and images for: Hepatitis B virus Core protein nuclear interactome identifies SRSF10 as a host RNA-binding protein restricting HBV RNA production
Source: PLoS Pathog. 2020 Nov 12;16(11):e1008593. doi: 10.1371/journal.ppat.1008593 (PMC7707522; doi:10.1371/journal.ppat.1008593)

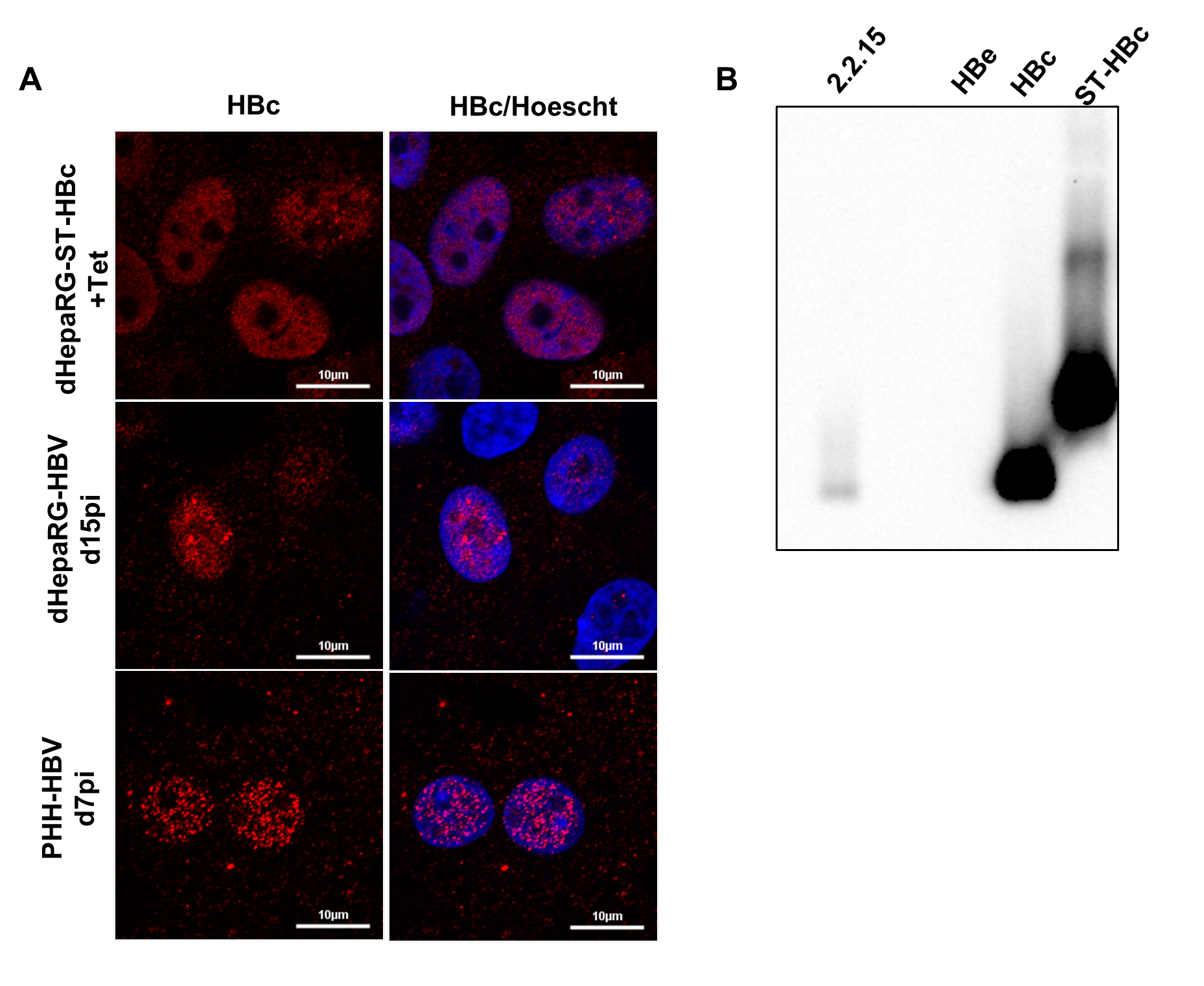

Supplement: S1 Fig — (A) Immunofluorescence (IF) of analysis of HBc localization in dHepaRG-TR-ST-HBc versus HBV-infected dHepaRG and PHH. (B) Intracellular HBV capsids, produced by the indicated cell lines, were analyzed by native gel electrophoresis followed by western blot with anti-HBc antibody. Lanes: 1. HepG2.2.15 2. dHepaRG-TR-HBe; 3. dHepaRG-TR-HBc; 4. dHepaRG-TR-ST-HBc. (TIF) [file ppat.1008593.s001.tif]

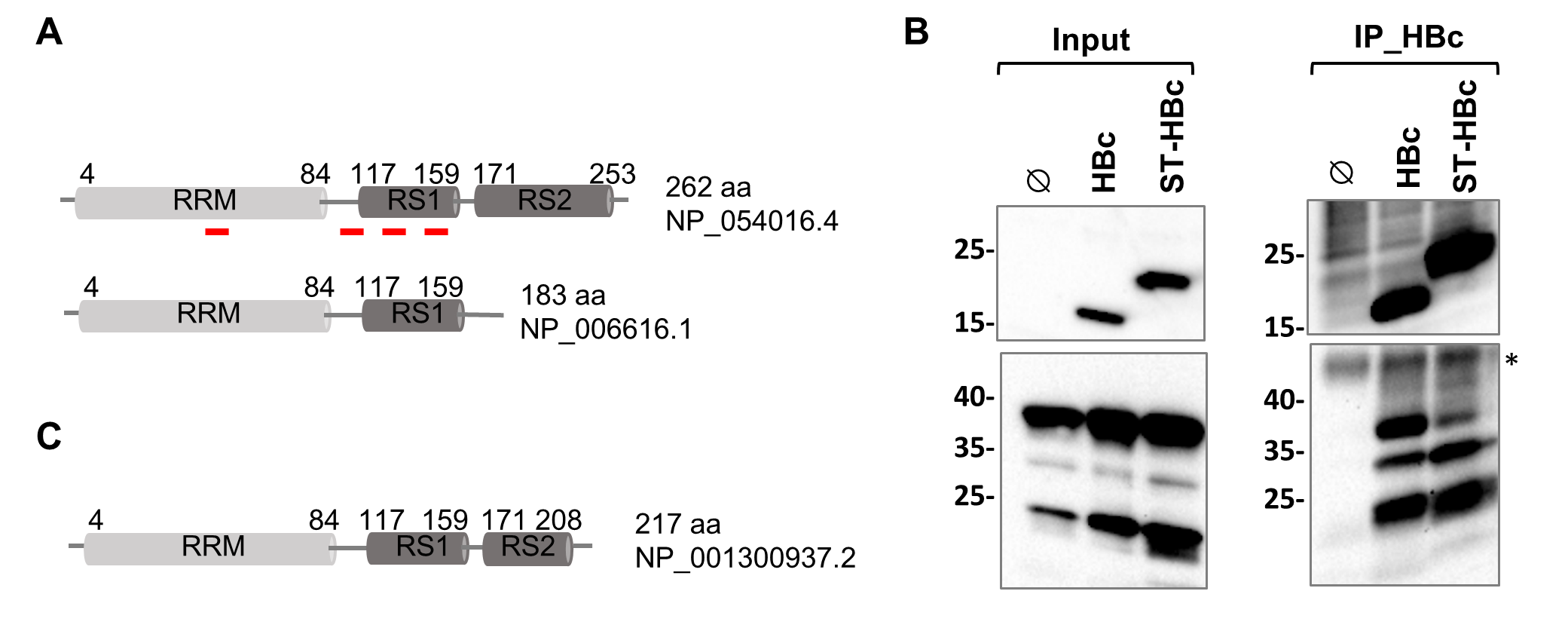

Supplement: S2 Fig — (A) Structure of the two major SRSF10 isoforms. These two SRSF10 variants that migrate at 37 and 20–22 KDa correspond to the two major SRSF10 isoforms detected by the anti-SRSF10 antibody (Ab77209). Occasionally additional bands with an intermediate size are visible as shown in panel B. The red lines correspond to the regions targeted by the siRNA. (B) HBc was immune-precipitated from nuclear extracts of dHepaRG-HBc (HBc), dHepaRG-ST-HBc (ST-HBc) and control dHepaRG (RG) cells induced with Tet for two days. Eluted proteins were analyzed by western blot using anti-HBc and anti-SRSF10 antibodies. The asterisk indicates the positions of IgG heavy chain. (C) Putative SRSF10 isoform migrating between 25 and 35 KDa. (TIF) [file ppat.1008593.s002.tif]

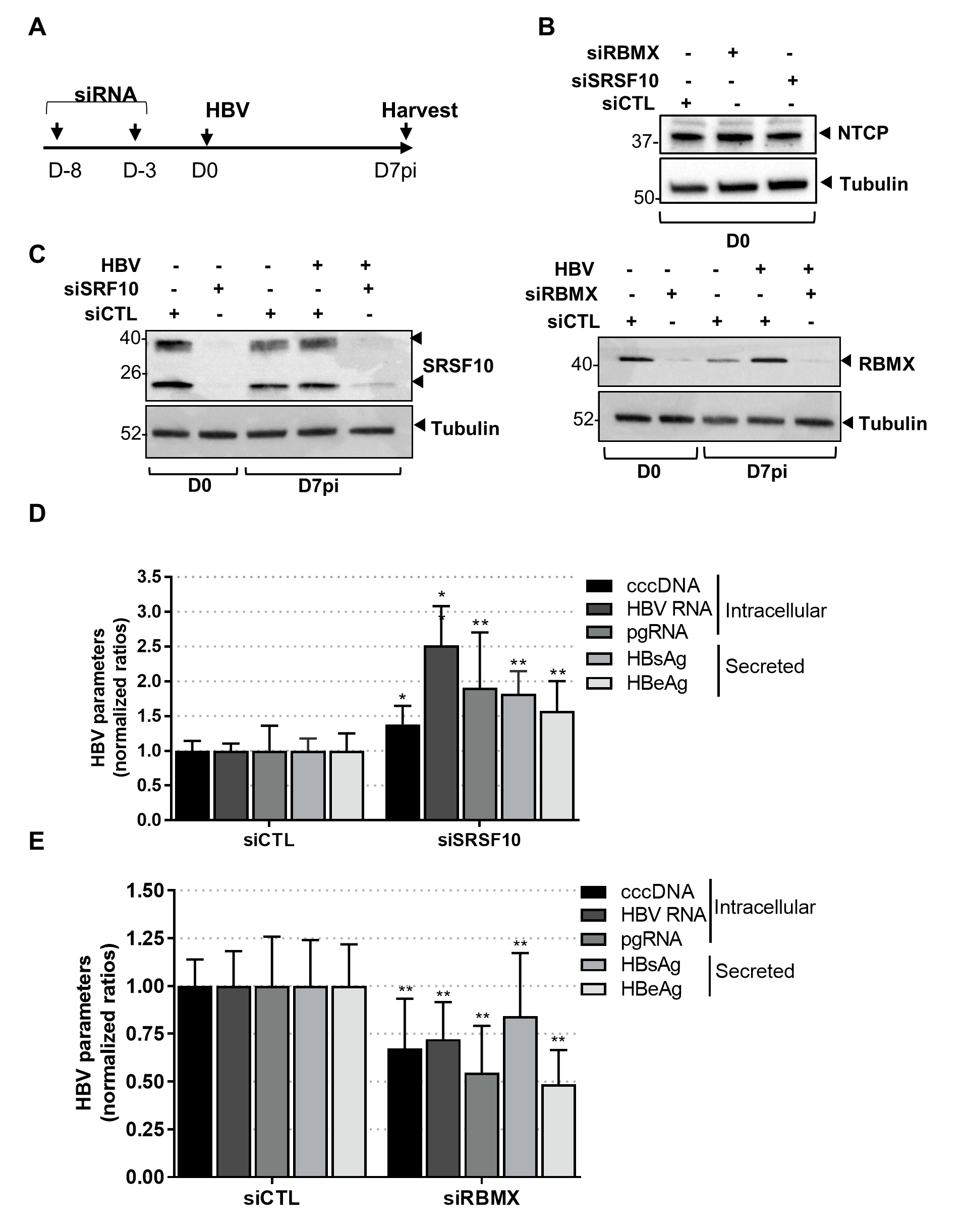

Supplement: S3 Fig — (A) Outline of the experimental protocol in dHepaRG cells: cells were transfected with siRNA targeting SRSF10 or RBMX or control siRNA (siCTL) and then infected with HBV (MOI of 250 vge/cell). (B) NTCP levels in siRNA transfected dHepaRG cells before HBV infection (D0). C. Western blot validations in cells secreted parameters measured at D7pi. Results are expressed as the mean normalized ratio +/- SD, between siSRSF10 or siRBMX and siCTL transfected cells, of 3 independent experiments, each performed in triplicate. (TIF) [file ppat.1008593.s003.tif]

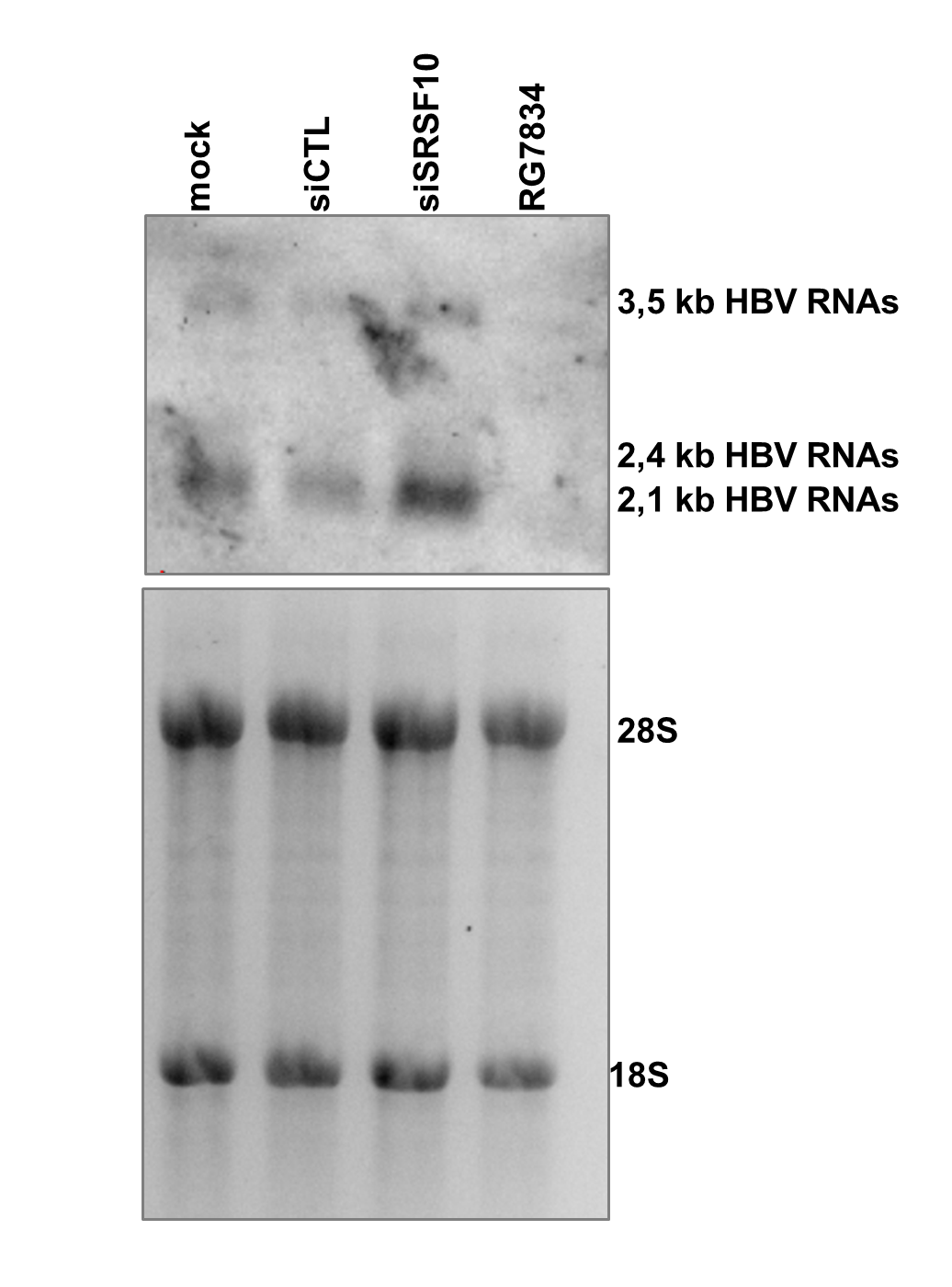

Supplement: S4 Fig — dHepaRG cells were transfected CTL or SRSF10 siRNA and infected with HBV as previously described (S3A Fig). Total RNA was extracted from cells at D7 pi and analyzed by Northern blot using HBV probes. (TIF) [file ppat.1008593.s004.tif]

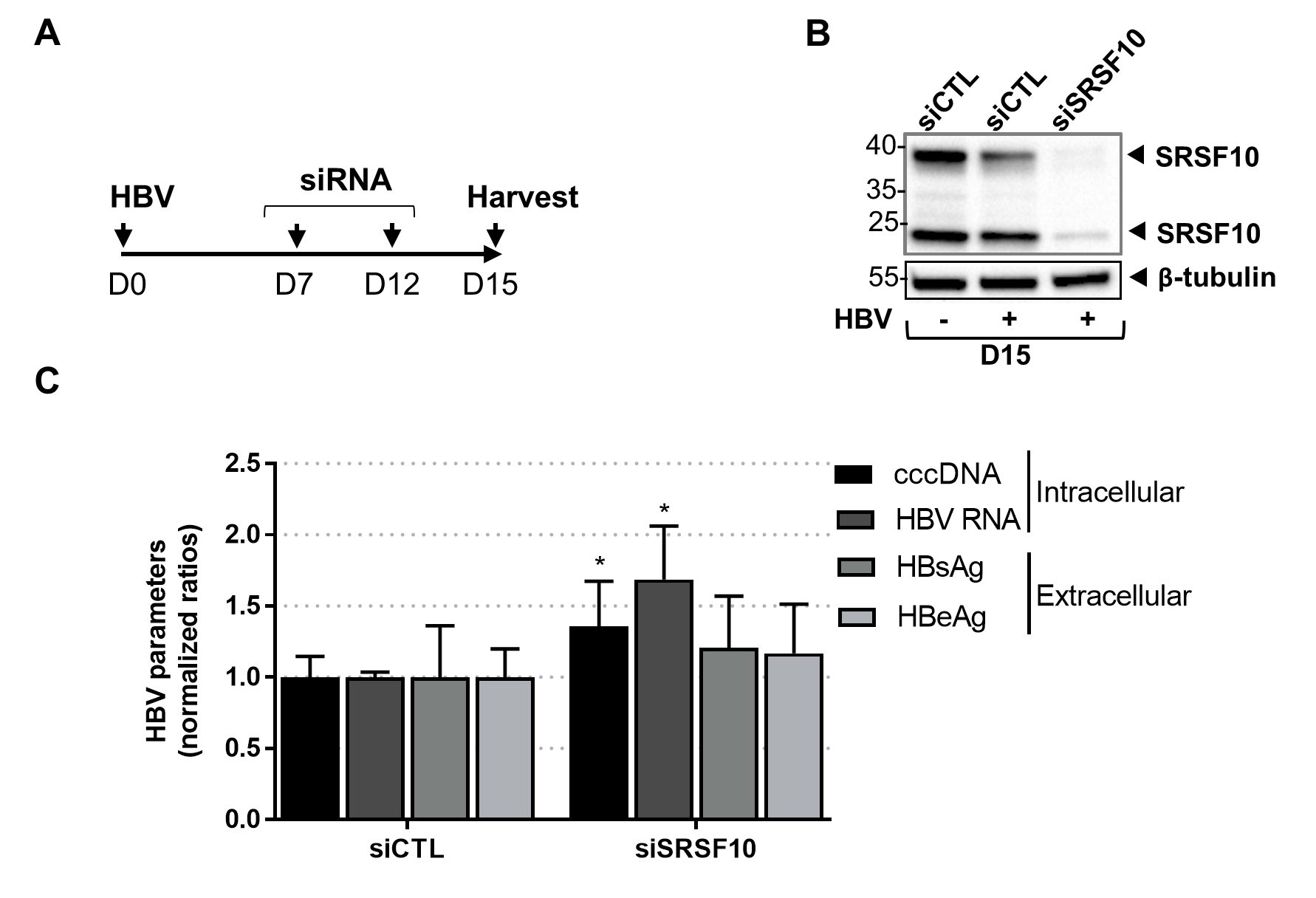

Supplement: S5 Fig — (A) Outline of the experimental protocol: dHepaRG cells were infected with HBV (MOI of 250 vge/cell) and then transfected twice with siRNA targeting SRSF10 or control siRNA (siCTL). Cells and supernatants were harvested at D15pi and analyzed to measure extracellular and intracellular HBV parameters. (B) Western blot validation of SRSF10 KD. (C) Effect of SRSF10 KD on intracellular and secreted HBV parameters. Results are expressed as the mean normalized ratio +/- SD, between siSRSF10 or siRBMX and siCTL transfected cells, of 3 independent experiments, each performed in triplicate. (TIF) [file ppat.1008593.s005.tif]

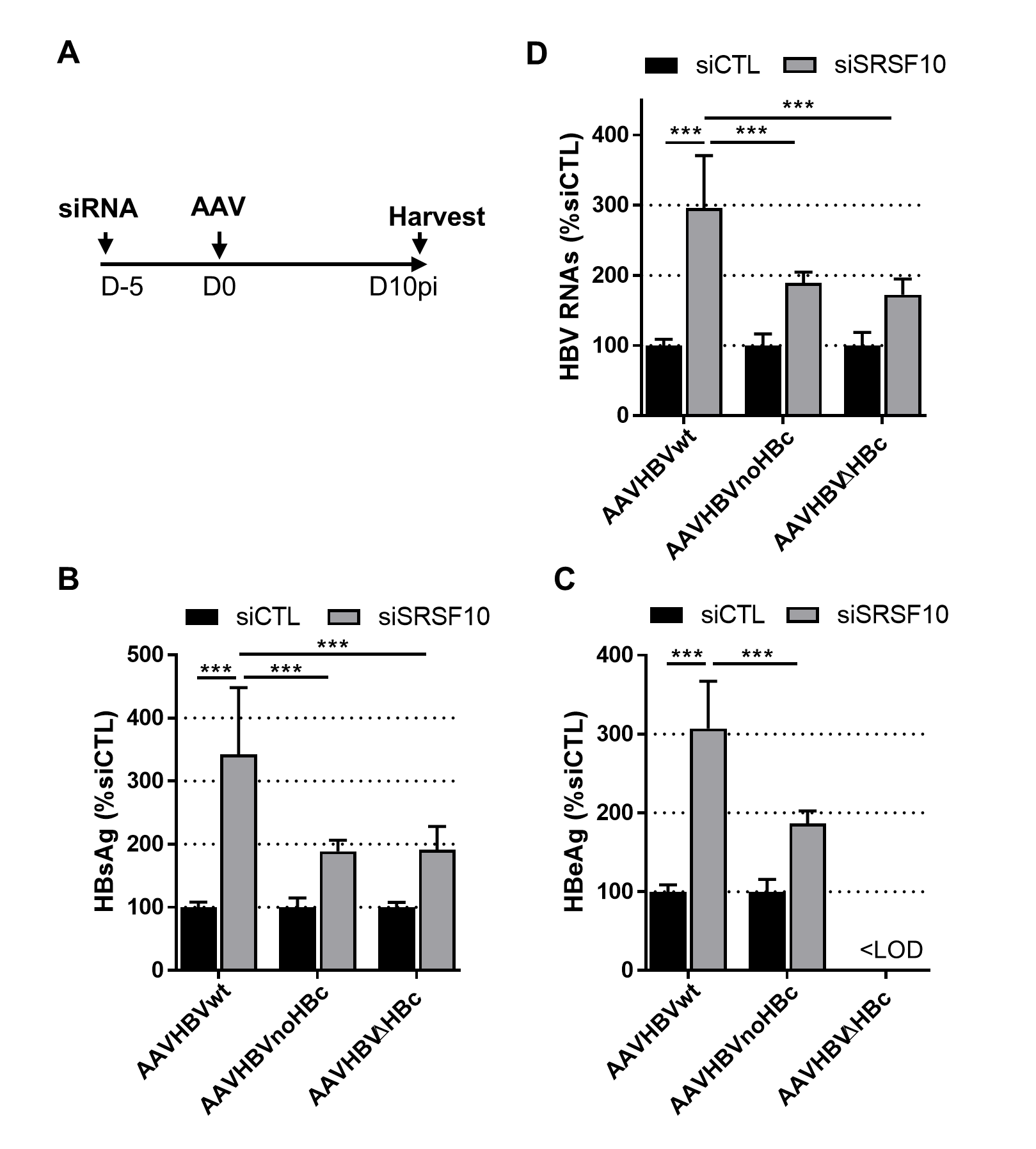

Supplement: S6 Fig — (A) dHepaRG cells were transfected with siRNA against SRSF10 or control siRNA and then transduced with AAV vectors containing either a wt (AAVHBVwt) or an HBc-deficient genome (AAVHBVnoHBc and AAVHBVΔHBc) at a MOI of 104 vge/cell. Secreted antigens and total RNAs were quantified 10 days later. Results are expressed as the mean normalized ratio +/- SD, between siSRSF10 and siCTL transfected cells, of 3 independent experiments, each performed in triplicate. (TIF) [file ppat.1008593.s006.tif]

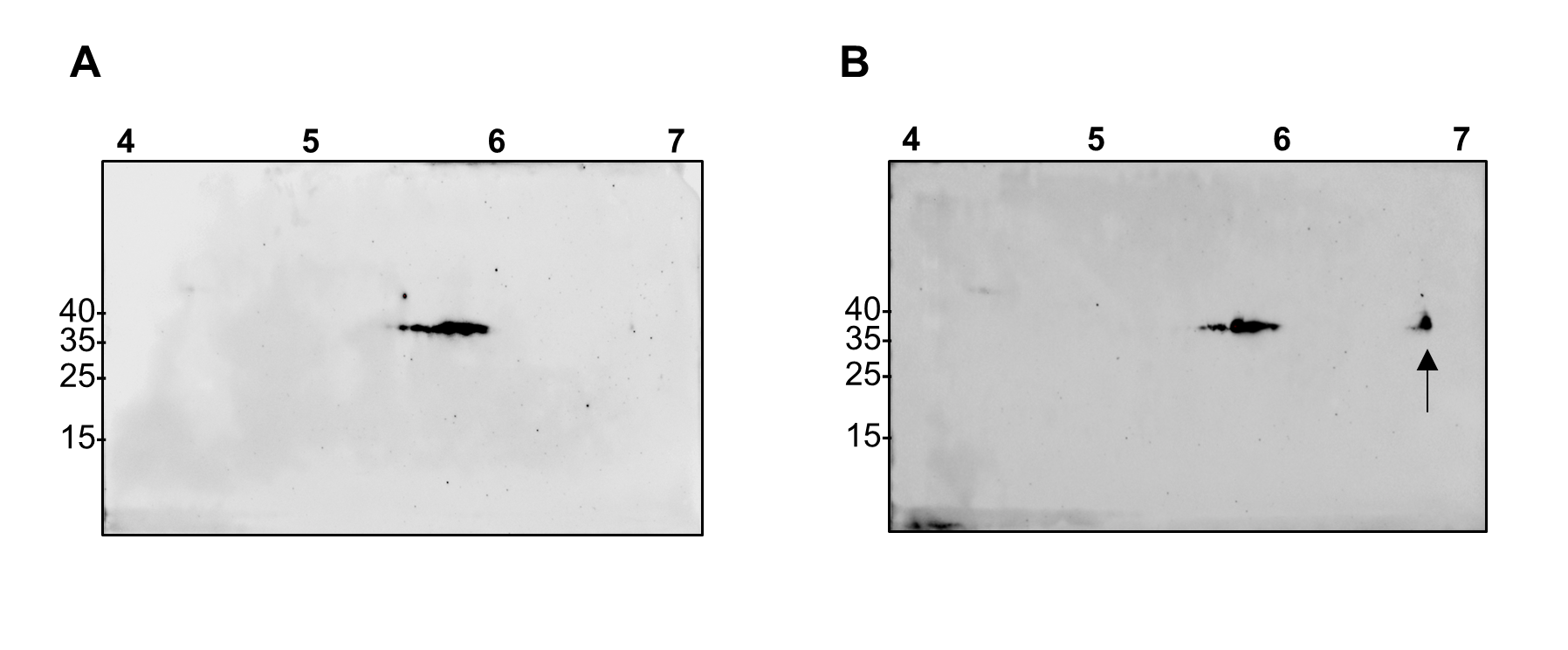

Supplement: S7 Fig — Nuclear extracts were prepared from dHepaRG cells either mock (A) or 1C8-treated (18 hrs at 20μM) (B) and separated by two-dimensional gel electrophoresis followed by western blot using an anti-SRSF10 antibody. Numbers on the top of the images indicate the pH gradient. Only the larger SRSF10 isoform of 37 KDa was visible under these conditions. The arrow indicates a hypo-phosphorylated isoform generated following 1C8 treatment. (TIF) [file ppat.1008593.s007.tif]

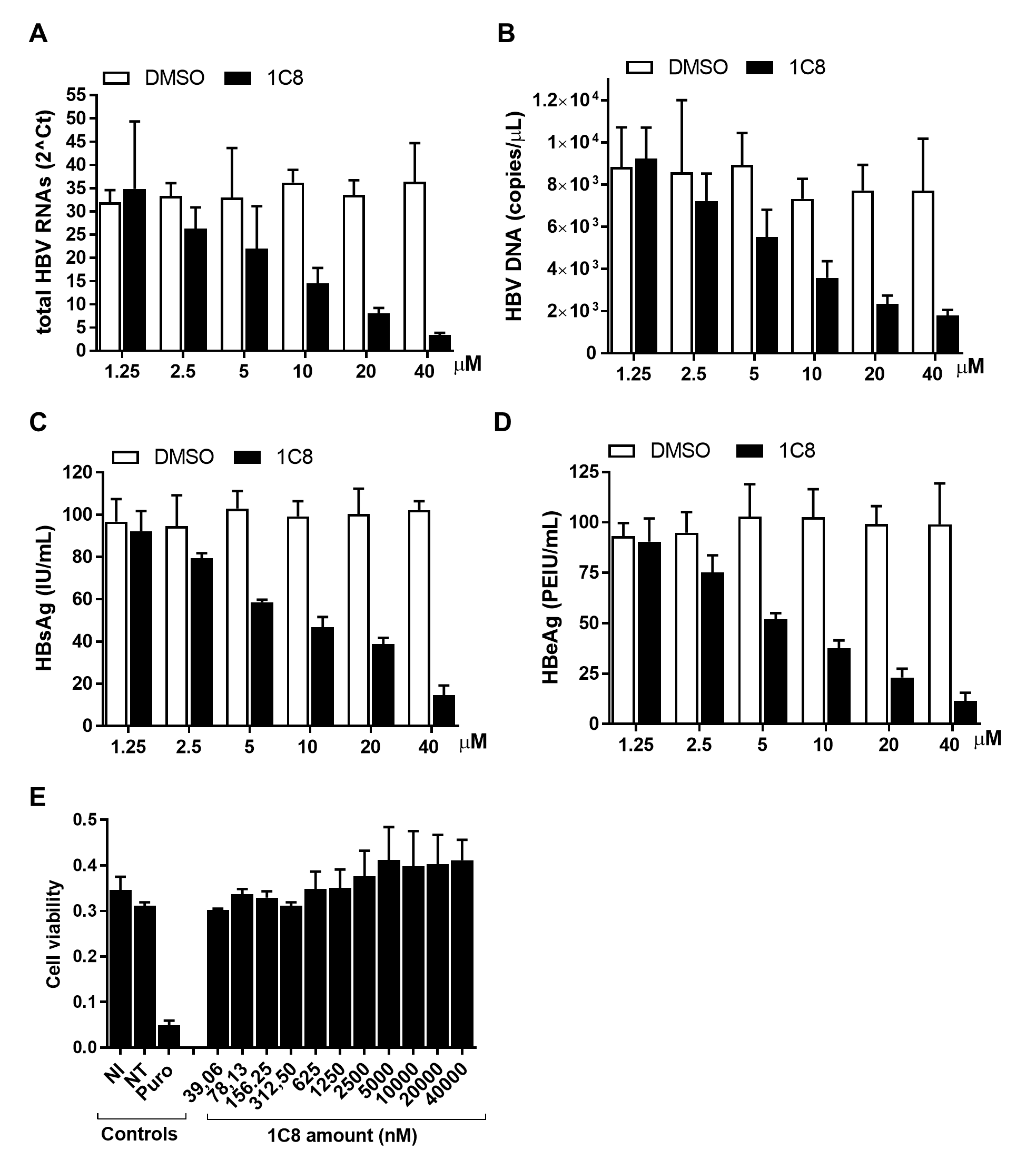

Supplement: S8 Fig — (A) to (D). Measure of 1C8 EC50 on HBV-infected dHepaRG. dHepaRG cells were infected with HBV (MOI of 250 vge/cell) for 7 days followed by three treatments with increasing concentration of 1C8. Total HBV RNAs (A), secreted HBV DNA (B), HBsAg (C) and HBeAg (D) were measured two days after the last treatment. Results are presented as the mean change in expression or secretion +/- SD of three independent experiments, each performed in triplicate. (E) Toxicity assay. Cell viability of dHepaRG cells treated with increasing concentrations of 1C8, was measured using the CellTiter-Glo Luminiscent Cell Viability Assay (Promega). Non-infected (NI) and HBV-infected dHepaRG cells treated with DMSO and puromycin were used as negative and positive controls, respectively. (TIF) [file ppat.1008593.s008.tif]

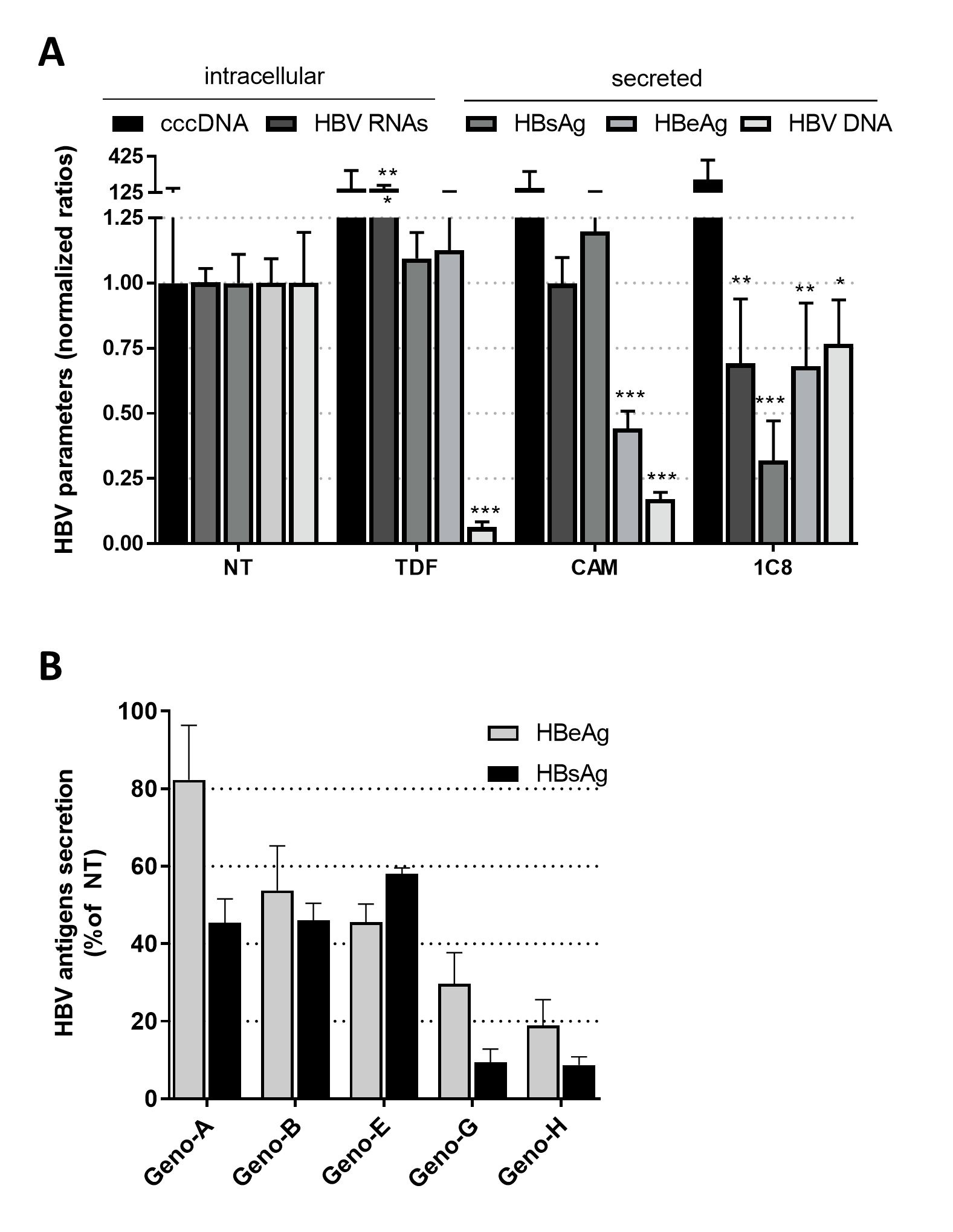

Supplement: S9 Fig — (A) Cells were infected with HBV genotype C (MOI of 100 vge/cell) and treated as indicated in Fig 5A. Treatments included, Tenofovir (TDF at 10 μM), a Core allosteric modulator (CAM at 10 μM) or 1C8 (10 μM). Intracellular and secreted HBV parameters were quantified 2 days after the last treatment. Results are expressed as the mean normalized ratio +/- SD between non-treated and treated cells of 2 independent experiments, each performed in triplicate. (B) Cells were infected with indicated HBV genotypes (MOI of 100 vge/cell) and either mock- or treated with 1C8 (10 μM). HBeAg and HBsAg were quantified by CLIA. Results are expressed as the mean normalized ratio +/- SD between non-treated and treated cells of a single experiment, with biological triplicates. (TIF) [file ppat.1008593.s009.tif]

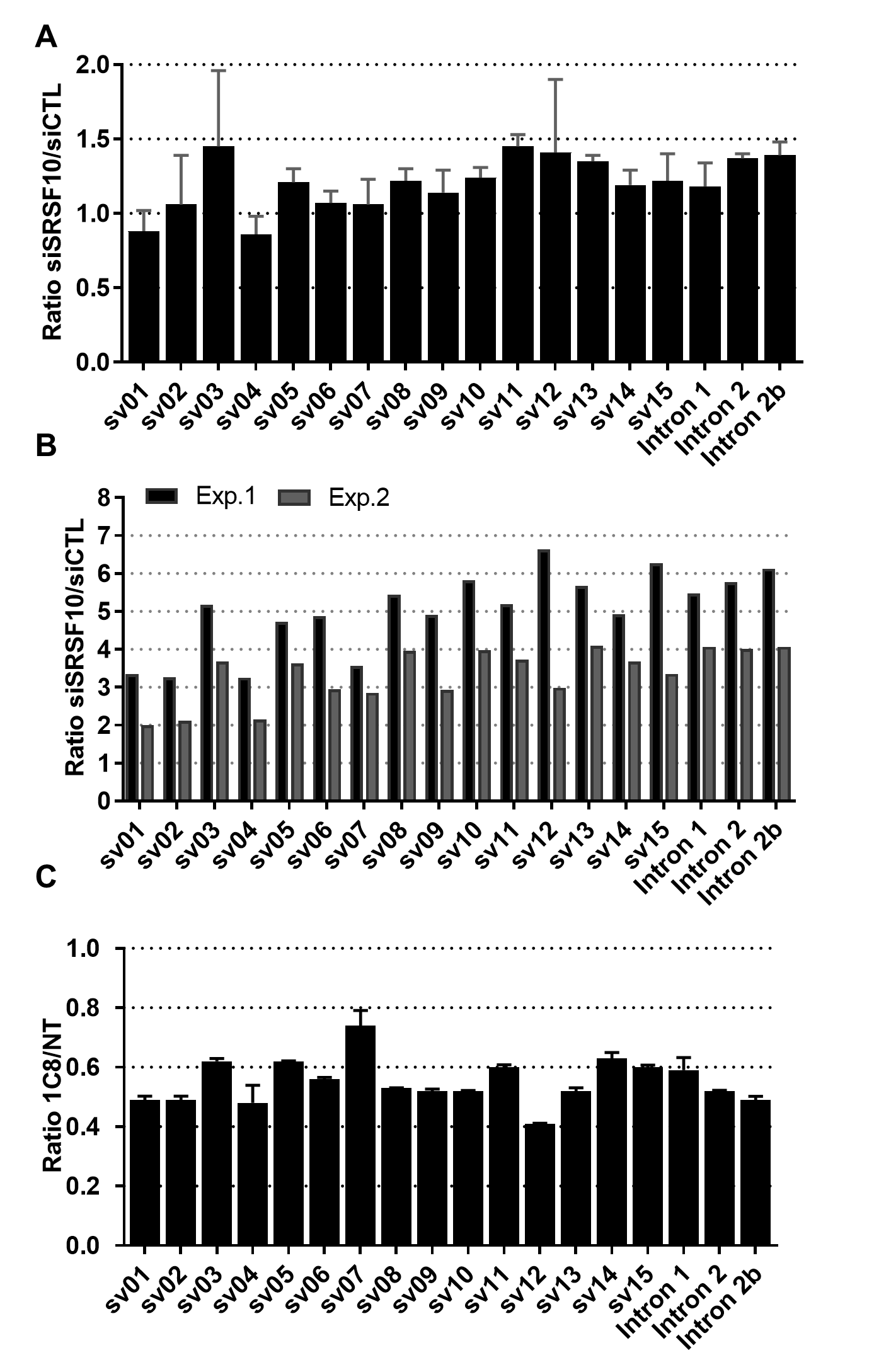

Supplement: S10 Fig — (A) and (B) Total RNA were extracted from dHepaRG (A) and PHH (B) transfected with siRNA following the previously described protocol (Fig 4A). (C) HBV-infected dHepaRG were treated with 1C8 as previously described (Fig 5B). HBV RNAs were analyzed by end-point RT-qPCR using sets of primers able to discriminate each spliced and unspliced form (see Methods section). Results are expressed as the mean ratio +/- SD between siSRSF10 and siCTL transfected cells of 3(A and C) or 2 (B) independent experiments. (TIF) [file ppat.1008593.s010.tif]
